# Control of synaptic vesicle release probability via VAMP4 targeting to endolysosomes

**DOI:** 10.1126/sciadv.abf3873

**Published:** 2021-04-30

**Authors:** Daniela Ivanova, Katharine L. Dobson, Akshada Gajbhiye, Elizabeth C. Davenport, Daniela Hacker, Sila K. Ultanir, Matthias Trost, Michael A. Cousin

**Affiliations:** 1 Centre for Discovery Brain Sciences, Hugh Robson Building, George Square, University of Edinburgh, Edinburgh EH8 9XD, Scotland.; 2 Muir Maxwell Epilepsy Centre, Hugh Robson Building, George Square, University of Edinburgh, Edinburgh EH8 9XD, Scotland.; 3 Simons Initiative for the Developing Brain, Hugh Robson Building, George Square, University of Edinburgh, Edinburgh EH8 9XD, Scotland.; 4 Newcastle University Biosciences Institute, Faculty of Medical Sciences, Newcastle upon Tyne NE2 4HH, UK.; 5 The Francis Crick Institute, 1 Midland Road, London NW1 1AT, UK.

## Abstract

Synaptic vesicle (SV) release probability (Pr), determines the steady state and plastic control of neurotransmitter release. However, how diversity in SV composition arises and regulates the Pr of individual SVs is not understood. We found that modulation of the copy number of the noncanonical vesicular SNARE (soluble *N*-ethylmaleimide–sensitive factor attachment protein receptor), vesicle-associated membrane protein 4 (VAMP4), on SVs is key for regulating Pr. Mechanistically, this is underpinned by its reduced ability to form an efficient SNARE complex with canonical plasma membrane SNAREs. VAMP4 has unusually high synaptic turnover and is selectively sorted to endolysosomes during activity-dependent bulk endocytosis. Disruption of endolysosomal trafficking and function markedly increased the abundance of VAMP4 in the SV pool and inhibited SV fusion. Together, our results unravel a new mechanism for generating SV heterogeneity and control of Pr through coupling of SV recycling to a major clearing system that regulates protein homeostasis.

## Teaser

VAMP4 is a molecular rheostat that adjusts neurotransmitter release to both the activity and degradative status of neurons.

## INTRODUCTION

The activity-dependent release of neurotransmitters at central synapses underpins brain function at the molecular, circuit, and behavioral level. Synaptic vesicle (SV) release probability (Pr), defined as the likelihood of an SV to fuse upon arrival of an action potential (AP), is one of the critical parameters in determining how synapses respond to changing patterns of activity. Pr is dynamically regulated by many interdependent factors, including presynaptic calcium homeostasis, the size of the readily releasable pool (RRP) of SVs, and SV heterogeneity (*1*).

SNARE (soluble *N*-ethylmaleimide–sensitive factor attachment protein receptor) proteins are essential components of the SV fusion machinery and therefore key regulators of Pr. The SV R-SNARE synaptobrevin-2 (syb2) is indispensable for evoked synchronous neurotransmission (*2*). However, SVs also contain other R-SNARE proteins, including many endosomal SNAREs (*3*), that may confer differences in SV fusogenicity, providing an attractive mechanism to alter Pr via dynamic control of their copy number on SVs.

The morphological uniformity of SVs is in notable contrast to their functional diversity. Therefore, SV heterogeneity arises primarily from differences in their molecular composition. However, how this heterogeneity is generated and how it affects presynaptic function remain largely unknown.

Different SV recycling modes have been proposed to define the functional characteristics of SVs by selectively tagging them with specific molecules (*4*). In support, activity-dependent bulk endocytosis (ADBE), which is triggered during intense neuronal activity, forms transient bulk endosomes from which vesicles enriched in the noncanonical SNARE vesicle-associated membrane protein 4 (VAMP4) are generated (*5*).

It is difficult, however, to envision how the few recycling pathways that operate in synaptic terminals (*6*) can be solely responsible for the modulation of SV protein copy number that determines SV heterogeneity. Therefore, it is likely that the dynamic incorporation and clearance of specific SV cargos from the SV pool can perform a key role in driving changes in the molecular composition and functional properties of SVs. The endolysosomal system, which contains organelles involved in intracellular trafficking and protein homeostasis, is the perfect candidate to mediate regulated clearance of specific SV proteins from the SV pool (*7*, *8*). The existence of a molecular and functional link between the local SV recycling and axonal endolysosomal trafficking machineries would enable coordinated regulation of presynaptic release properties and global quality control mechanisms to optimally support long-term neuronal health and functionality.

In this study, we provide compelling evidence for such a cross-talk in the control of neurotransmitter release. We reveal that modulation of the copy number of VAMP4 in the SV pool is a key determinant of Pr. VAMP4-enriched SVs have reduced fusogenicity, due to an inefficient interaction with the canonical synaptic Q-SNAREs. They also exhibit high axonal turnover rates, with a substantial pool transported retrogradely through the endolysosomal system. Inhibition of either ADBE or endolysosomal trafficking and function profoundly increased the abundance of VAMP4 in nerve terminals and consequently inhibited SV fusion. Disruption of adaptor protein 1 (AP1) function, previously shown to have a redundant role in SV recycling (*9*), impairs cargo sorting to endolysosomes, resulting in synaptic enrichment and a reduced turnover of VAMP4. Conversely, *Vamp4* knockout (KO) nerve terminals displayed enhanced Pr and an inability to sustain synaptic facilitation. The inhibition of SV fusion observed in response to suppression of axonal endolysosomal flux is absent in *Vamp4* KO synapses, confirming a key role for VAMP4 in translating lysosomal dysfunction into altered presynaptic release properties. Last, proteomic profiling revealed a reciprocal control of the endolysosomal and SV fusion machineries by VAMP4, with a selective de-enrichment of endolysosomal proteins in isolated VAMP4 KO nerve terminals. Our findings therefore uncover the SV recycling and axonal endolysosomal machineries as a continuum and postulate VAMP4 as an essential component in a mechanism that integrates both input-specific activity patterns and cell-wide alterations in neuronal proteostasis to tune Pr.

## RESULTS

### Synaptic accumulation of VAMP4 lowers SV fusion competence

The R-SNARE VAMP4 has established cellular roles in endosome–to–trans-Golgi network (TGN) transport in non-neuronal cells (*10*, *11*). To reveal its neuronal function, we examined its expression on fixed brain sections from 3- to 4-month-old mice using immunohistochemistry (IHC). Dense VAMP4 labeling was detected throughout the brain, suggesting a potential widespread role (fig. S1A). In primary cultures of hippocampal neurons, prominent immunofluorescence for VAMP4 was present in neuronal cell bodies, closely mirroring the VAMP4 interaction partner AP1 (*12*) that is localized to TGN and endosomes (fig. S1B). In addition, discrete punctate immunoreactivity for VAMP4 was detected in presynaptic terminals, albeit at a lower level to cell bodies (fig. S1C). Biochemical fractionation of adult mouse brain tissue confirmed the high enrichment of VAMP4 in both cell body organelles and SVs (fig. S1, D and E) (*13*). Therefore, VAMP4 is widely expressed in mammalian brain, with considerable presynaptic expression.

**Fig. 1:**
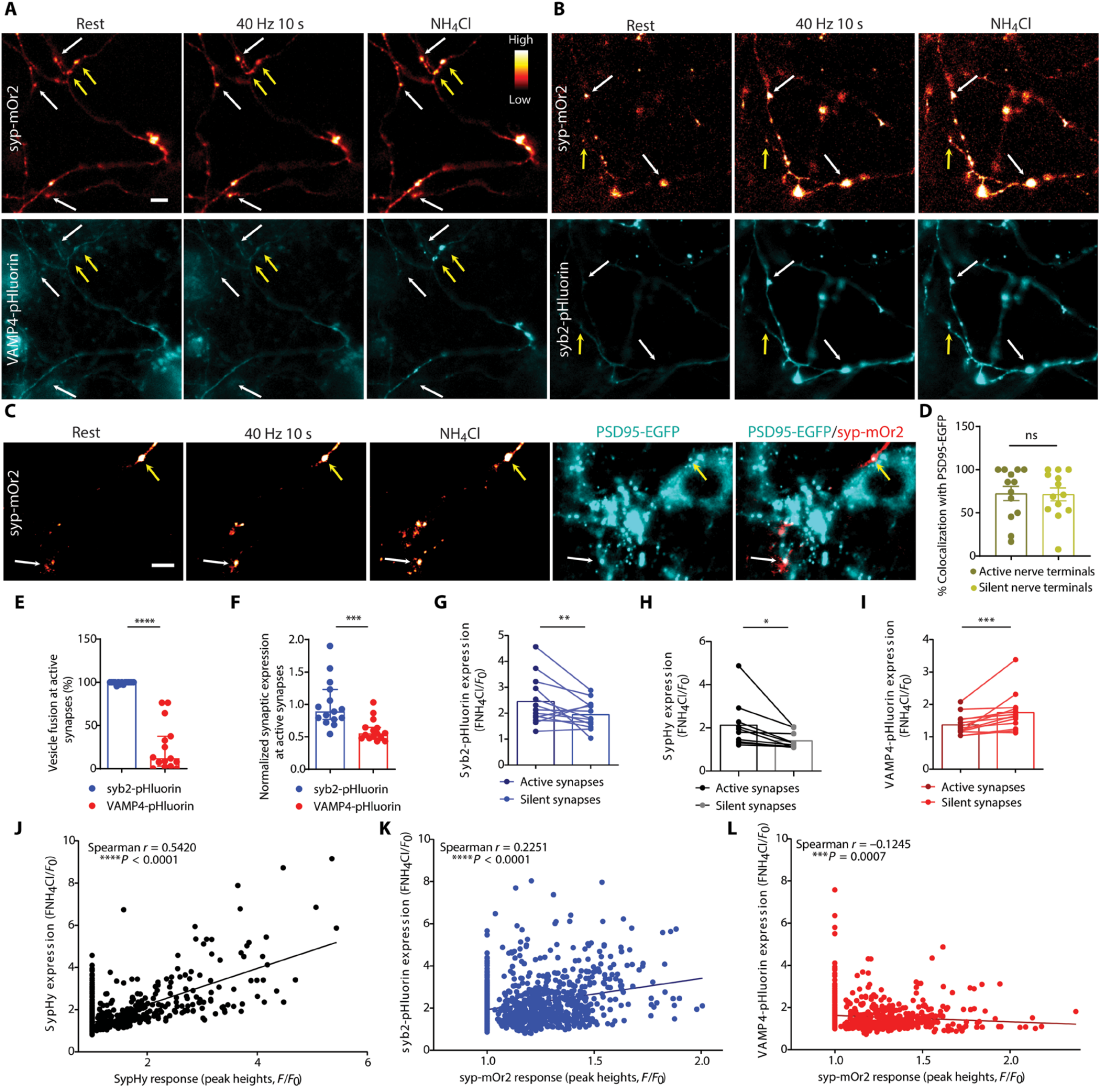
Synaptic accumulation of VAMP4 lowers SV fusion competence. Primary cultures of hippocampal neurons were cotransfected with syp-mOr2 and either VAMP4-pHluorin or syb2-pHluorin. Neurons were stimulated with 400 APs (40 Hz) and pulsed with NH_4_Cl imaging buffer after 200 s. (**A** and **B**) Representative images of the fluorescent response of syp-mOr2 (A and B), VAMP4-pHluorin (A), and syb2-pHluorin (B) are displayed at rest, during stimulation, or during exposure to NH_4_Cl [white arrows, nerve terminals that display a syp-mOr2 response (active synapses); yellow arrows, those that do not (silent synapses)]. Scale bar, 5 μm. (**C** and **D**) Hippocampal neurons transduced with PSD95-EGFP after a prior transfection with syp-mOr2 were subjected to an identical protocol as above. (C) Representative images of syp-mOr2 and PSD95-EGFP at rest, during stimulation, or during NH_4_Cl exposure (white arrows, active synapses; yellow arrows, silent synapses). Scale bar, 5 μm. (D) Number of active or silent syp-mOr2 puncta colocalizing with PSD95-EGFP (*n* = 13 coverslips from four independent preparations, *P* = 0.82, Mann-Whitney test). (**E** and **F**) Number of synapses with fusion events reported by pHluorin (E) or the expression level of pHluorin at synapses that show an activity-dependent syp-mOr2 response (F). *n* = 15 coverslips, each from four independent preparations, ****P < 0.001 and ***P = 0.001, Mann-Whitney test. (**G** to **I**) Expression level of syb2-pHluorin (G), sypHy (H), and VAMP4-pHluorin (I) at active and silent synapses. *n* = 10 (G) or *n* = 14 (H and I) coverslips from four independent preparations, ***P = 0.0005, **P = 0.002, and *P = 0.039, either Wilcoxon matched-pairs signed-rank test (G and I) or paired *t* test (H). (**J** to **L**) Correlation between the extent of the sypHy response with the expression of sypHy (J) or the syp-mOr2 response with the expression of syb2-pHluorin (K) and VAMP4-pHluorin (L). ns, not significant.

VAMP4 is proposed to perform a role in asynchronous release at inhibitory synapses (*14*). However, asynchronous release is only dominant at specialized synapses in the central nervous system (CNS) (*15*, *16*), which contrasts with the widespread distribution of VAMP4 in mammalian brain. At most synapses, SV fusion is tightly temporarily coupled to APs (synchronous release), which allows rapid information transfer within the CNS. Therefore, we sought to examine whether VAMP4 had a more ubiquitous function in synchronous SV fusion. We first visualized SV fusion in real time via the expression of genetically encoded reporters in primary cultures of hippocampal neurons. These probes report the pH of their immediate environment via a pH-sensitive fluorescent moiety fused to the lumenal domain of an SV protein (*17*). SV fusion is reported as increase in fluorescence, as the reporter transitions from the acidic SV lumen to the neutral extracellular environment on the cell surface. Neurons were transfected with syp-mOr2 [synaptophysin tagged with the pH-sensitive, red-shifted mOrange2; (*18*)] and either VAMP4-pHluorin or the canonical SV R-SNARE syb2-pHluorin. The cotrafficking of these reporters was monitored during a challenge with a train of APs at high frequency (40 Hz, 10 s) using time-lapse imaging in live neurons (Fig. 1, A and B). Synapses that displayed an activity-dependent syp-mOr2 response were classified as active synapses (white arrows in Fig. 1, A and B). Only 25% of VAMP4-pHluorin molecules, on average, visited the cell surface at these active synapses during stimulation (Fig. 1, A and E). In contrast, the syb2-pHluorin response tightly tracked that of syp-mOr2 and reported SV fusion events at nearly 100% of active synapses (Fig. 1, B and E). Notably, VAMP4-pHluorin expression at active synapses was significantly lower when expressed relative to that of syb2-pHluorin (Fig. 1F). This suggests that increased expression of VAMP4 may limit SV fusion during neuronal activity.

The following set of experiments corroborated this hypothesis. A fraction of synapses (~35% of all synapses per field of view) that expressed syp-mOr2 displayed no evoked SV fusion events (silent synapses; yellow arrows in Fig. 1, A and B). This was also the case in neurons transfected with sypHy (synaptophysin-pHluorin) alone. Colocalization analyses of syp-mOr2 clusters and the excitatory postsynaptic marker PSD95–EGFP (enhanced green fluorescent protein) revealed ~75% overlap between the pre- and postsynaptic marker for both active and silent synapses (Fig. 1, C and D). Furthermore, post hoc colocalization analyses in fixed neuronal cultures reported a similar degree of colocalization between VAMP4-pHluorin, syb2-pHluorin, and endogenous PSD95 (fig. S1, F and G). This confirms that most of the presynaptically silent synapses are structurally complete, bona fide synapses. Notably, expression of VAMP4-pHluorin at these silent synapses was significantly higher than at active synapses from the same neurons (Fig. 1, A and I). Conversely, expression of sypHy and syb2-pHluorin at silent synapses was reduced when compared to active synapses (Fig. 1, B, G, and H). Further analysis revealed a highly significant positive correlation between the amplitudes of the evoked synaptic responses (sypHy or syp-mOr2 peak heights) and the levels of expression of sypHy and syb2-pHluorin at individual synapses (Fig. 1, J and K). In contrast, a significant negative correlation was observed between the expression of VAMP4-pHluorin and the synaptic responses (Fig. 1L). Together, these findings suggest that synaptic enrichment of VAMP4 reduces SV fusion and reveals VAMP4 as a potential negative regulator of Pr. Furthermore, they imply that synaptic activity is required to maintain a low expression of VAMP4 in nerve terminals, revealing a potential input-specific feedback mechanism for synaptic facilitation.

### VAMP4 does not form SNARE complexes efficiently during synaptic activity

One potential mechanism via which VAMP4 could negatively regulate Pr is to interfere with syb2-mediated SNARE complex assembly. The amino acid sequence of the VAMP4 SNARE motif is relatively similar to syb2 (62%); however, important sequence variations between them (fig. S2A) (*19*) may subtly alter the kinetics of SNARE complex formation. Therefore, we next determined whether VAMP4 could form an SNARE complex with plasma membrane Q-SNAREs in response to neuronal activity with the same efficiency as syb2.

**Fig. 2:**
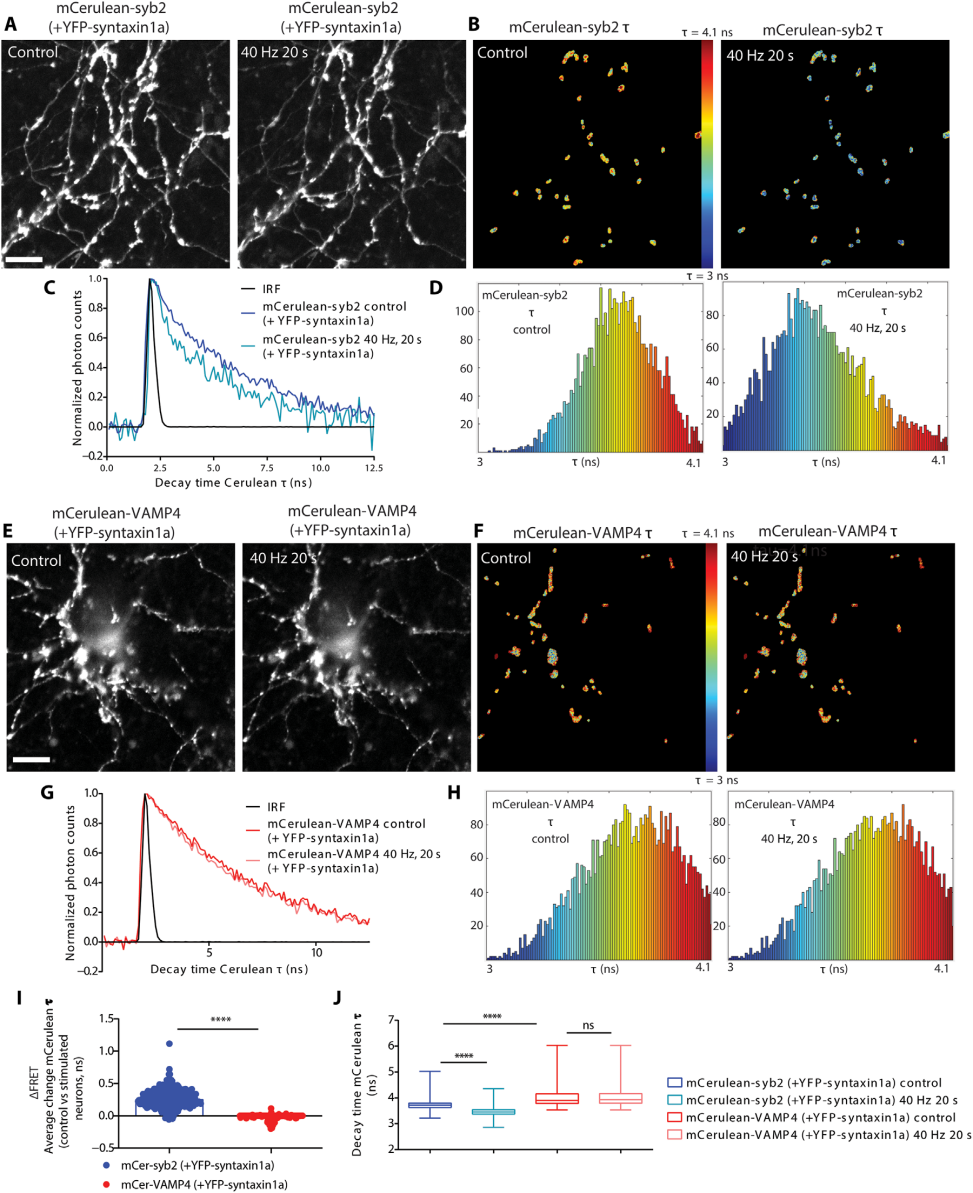
VAMP4 does not form SNARE complexes efficiently during synaptic activity. Primary cultures of hippocampal neurons were cotransfected with YFP-syntaxin1a and either mCer-syb2 or mCer-VAMP4 and stimulated with 800 APs (40 Hz) while monitoring the fluorescence lifetime (τ) of the mCer donor. (**A**, **B**, **E**, and **F**) Representative images of the fluorescence intensity (A and E) or τ [using pseudo-color to report τ (B and F)] of either mCer-syb2 (A and B) or mCer-VAMP4 (E and F) before (control) or during (40-Hz, 20-s) stimulation. Scale bars, 10 μm. (**C**, **D**, **G**, and **H**) Normalized fluorescence decay (C and G) and τ (D and H) of either mCer-syb2 (C and D) or mCer-VAMP4 (G and H) from the experiments in (B) and (F), respectively. (**I**) ΔFRET indicates the average change in mCer-syb2 or mCer-VAMP4 τ before and during stimulation. (**J**) Average τ is displayed for all conditions. *n* = 301 nerve terminals from 7 independent experiments (syb2) and *n* = 256 nerve terminals from 17 independent experiments (VAMP4), Mann-Whitney test (I) and two-way analysis of variance (ANOVA) with Dunn’s multiple comparison test (J), ****P < 0.001. IRF, instrument response function.

To achieve this, we used fluorescence lifetime imaging (FLIM) to measure the Förster resonance energy transfer (FRET) at synapses in live neurons expressing YFP (yellow fluorescent protein)–tagged syntaxin1a and either mCerulean (mCer)–tagged syb2 or VAMP4 (Fig. 2, A and E). FRET occurs when the physical separation of two fluorophores with overlapping emission/excitation spectra is less than 10 nm, resulting in a decrease in the time that the donor (such as mCer) spends in an excited state. In our settings, an interaction with YFP-syntaxin1a will be detected as a decrease in the fluorescence lifetime of either mCer-VAMP4 or mCer-syb2. FLIM images of the same presynaptic terminals at rest and during high-frequency stimulation (40 Hz, 20 s) were acquired, and the fluorescence lifetime of mCer was calculated (Fig. 2, B, C, F, and G). The average mCer fluorescence lifetime at synapses of neurons cotransfected with mCer-syb2 and YFP-syntaxin1a was significantly lower during stimulation, suggesting an increased FRET (Fig. 2, B, D, I, and J). This is consistent with the engagement of these two molecules in an SNARE complex in response to synaptic activity. In contrast, a negligible reduction in the mCer fluorescence lifetime was detected during stimulation in neurons cotransfected with mCer-VAMP4 and YFP-syntaxin1a (Fig. 2, F to J). No difference was observed between resting and stimulated conditions in neurons transfected only with the donor, mCer-syb2, excluding nonspecific effects on the FRET measurements (fig. S2, B to F). FLIM-based FRET is independent of the donor concentration, since the fluorescence lifetime is an intrinsic characteristic of each fluorophore; however, it can be affected by the acceptor concentration. However, no difference in YFP-syntaxin1a expression was detected between mCer-VAMP4– and mCer-syb2–enriched synapses, ruling out an effect of variable acceptor concentrations on the FRET readouts (fig. S2, G and H). Thus, the inability of VAMP4 to interact efficiently with the canonical synaptic Q-SNAREs in an activity-dependent manner may explain why its increased abundance on SVs renders them less fusion competent.

### VAMP4 is retrieved constitutively from axons through the endolysosomal system

Increased neuronal activity results in the accelerated degradation of presynaptic proteins through the endolysosomal system, to prevent accumulation of damage during repetitive SV recycling (*20*, *21*). The SVs that have participated in recycling display decreased fusogenicity (*21*) and may form what has been termed as the “resting pool” of SVs that are inaccessible to synaptic stimulation. We suggest that accumulation of VAMP4 on SVs, which reduces their fusogenicity, marks an inactive SV subpopulation for elimination from synapses. The reduced fusion competence of these vesicles may be advantageous for long-range trafficking, as it will prevent their fusion in transit. We therefore hypothesized that VAMP4 should have a higher synaptic turnover rate than other SV proteins, since it will be continuously expelled from axons.

**Fig. 3:**
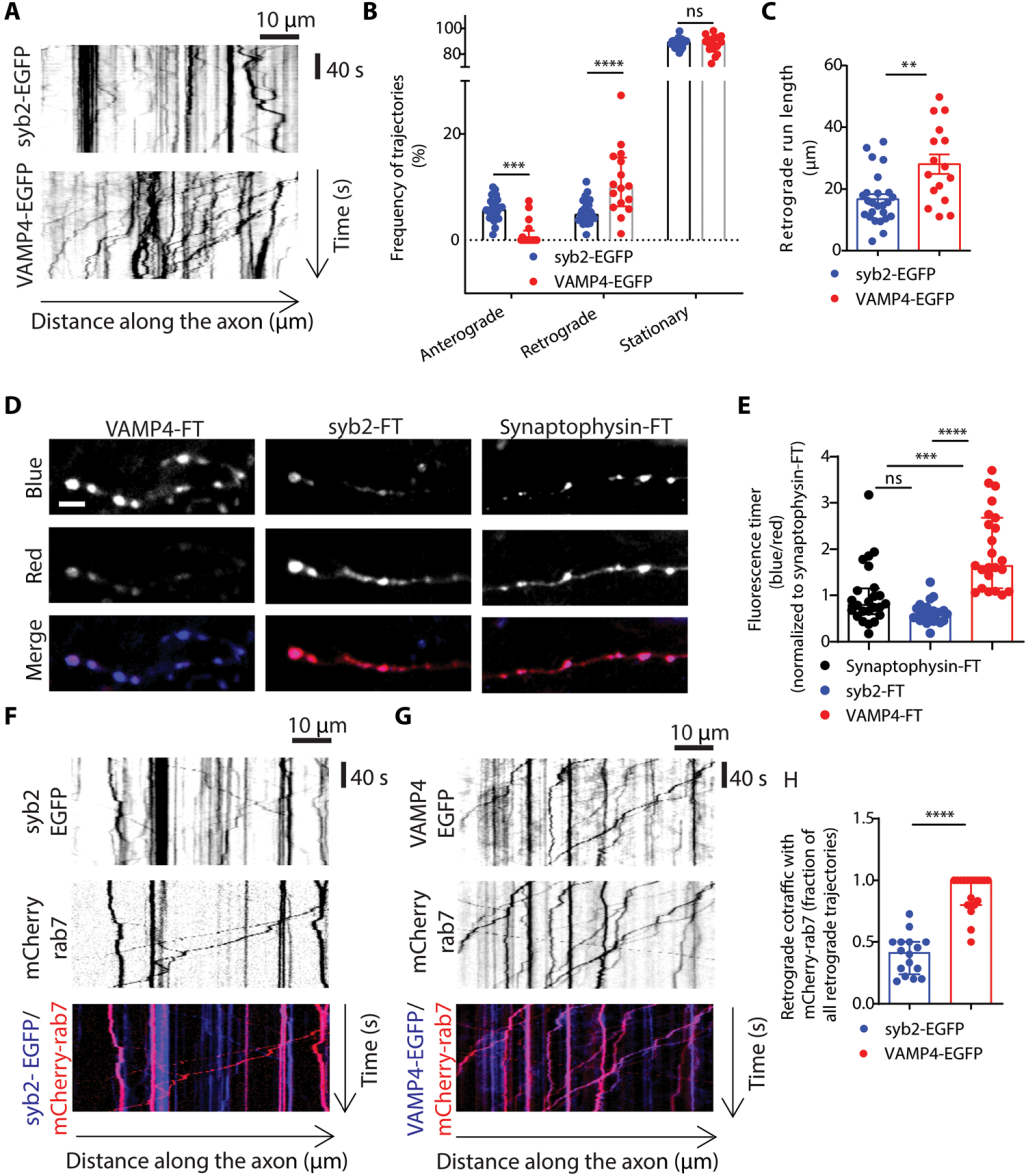
VAMP4 is retrieved constitutively from axons through the endolysosomal system. (**A**) Representative kymographs display the axonal mobility and directionality of traffic of VAMP4-EGFP and syb2-EGFP. (**B** and **C**) Frequency of anterograde, retrograde, or stationary trajectories (B) or the run length of retrograde trajectories (C) for both syb2-EGFP and VAMP4-EGFP. *n* = 25 (syb2) or *n* = 16 coverslips (VAMP4) from four independent preparations, ****P < 0.001 and **P = 0.0002, two-way ANOVA with Fisher’s least significant difference (B), and **P = 0.0012, unpaired *t* test (C). (**D** and **E**) Hippocampal neurons were transfected with syb2, synaptophysin, or VAMP4 tagged with an FT protein. (D) Representative images display FT protein fluorescence in either the blue (new protein), red (old protein), or merged channel. Scale bar, 5 μm. (E) Blue/red ratio of FT proteins. *n* = 24 (syb2 and synaptophysin) or *n* = 23 cells (VAMP4) from four independent preparations, ****P < 0.001 and ***P = 0.0004, Kruskal-Wallis test with Dunn’s multiple comparisons test. (**F** to **H**) Hippocampal neurons were transfected with mCherry-rab7 and either syb2-EGFP or VAMP4-EGFP. (F and G) Kymographs show the retrograde cotrafficking of syb2-EGFP and VAMP4-EGFP with mCherry-rab7. (H) Fraction of retrograde trajectories where syb2-EGFP or VAMP4-EGFP cotraffic with mCherry-rab7. *n* = 16 (syb2) or *n* = 18 (VAMP4) coverslips from four independent preparations, ****P < 0.001, Mann-Whitney test.

To evaluate VAMP4 axonal turnover, we first compared the overall mobility and trafficking pattern of VAMP4-EGFP in remote axonal regions to the trafficking behavior of syb2-EGFP using kymograph analysis (Fig. 3A). We found that there is a highly mobile pool of VAMP4-EGFP in axons of mature neurons [13 to 15 days in vitro (DIV)] that exhibits a clear retrograde transport bias in comparison to syb2-EGFP (Fig. 3, A and B). We also revealed an increased processivity of retrograde axonal transport for VAMP4-EGFP vesicles, highlighted by the significantly higher average run length of these vesicles when compared to syb2-EGFP (Fig. 3C). Therefore, we conclude that VAMP4-containing vesicles exhibit increased clearance from axons during basal activity.

We next determined the impact of increased neuronal activity on the axonal trafficking of VAMP4 and syb2. Synaptic stimulation (40 Hz, 10 s) significantly reduced the mobile fraction of syb2-EGFP vesicles and increased the number of stationary particles (fig. S3, A to D). It also slightly reduced the average retrograde run length of VAMP4-EGFP (fig. S3H). These results highlight that synaptic activity has a global impact on axonal transport, in agreement with previous observations showing that synaptic stimulation reduces the mobility of both membranous organelles and single molecular cargos (*22*–*25*). However, enhanced neuronal activity did not affect either the directionality or the overall number of mobile VAMP4-EGFP vesicles (fig. S3, E to G), supporting the notion that these vesicles are constructed to maintain long-distance trafficking between synaptic terminals and neuronal cell bodies.

SV cargos typically display low turnover rates at synapses (*26*). However, the enhanced retrograde flux of VAMP4 from synapses suggests that its lifetime in nerve terminals may be lower than other SV proteins. To directly compare the presynaptic turnover rate of VAMP4 with that of other SV proteins, we tagged VAMP4, syb2, and synaptophysin with the mCherry-derived monomeric fluorescent timer (FT)–slow (*27*). This fluorescent protein changes fluorescence from blue to red over time, and therefore, the ratio of blue-to-red signal provides an accurate estimate of the relative age of the tagged protein. When assessed, the blue-to-red ratios of both syb2-FT and synaptophysin-FT were comparable and relatively low, consistent with long synaptic lifetimes (Fig. 3, D and E). In contrast, the red form of VAMP4-FT was barely detectable in nerve terminals, and it exhibited a twofold increase in its blue-to-red ratio, indicative of a fast presynaptic turnover rate (Fig. 3, D and E).

The small guanosine triphosphatase (GTPase) rab7 couples presynaptic endocytosis to fast retrograde axonal transport and is required for maturation of late endosomes, autophagosomes, and lysosomes (here termed collectively as endolysosomes) (*28*). To determine the nature of VAMP4 retrograde carriers in axons, we examined the retrograde cotrafficking of VAMP4-EGFP and syb2-EGFP with mCherry-tagged rab7, using dual-color time-lapse imaging in live neurons. Whereas less than 50% of syb2-EGFP vesicles cotrafficked with mCherry-rab7 in the retrograde direction, almost 100% of VAMP4-EGFP vesicles displayed retrograde cotrafficking (Fig. 3, F to H). This suggests that VAMP4 retrograde carriers are a part of the axonal endolysosomal compartment. Together, these results show that VAMP4 has a high presynaptic turnover rate for an SV cargo and a substantial pool of VAMP4-enriched vesicles is constitutively trafficked to neuronal cell bodies through the endolysosomal system.

### AP1-mediated sorting of VAMP4 to endolysosomes regulates its abundance in the SV pool

**Fig. 4:**
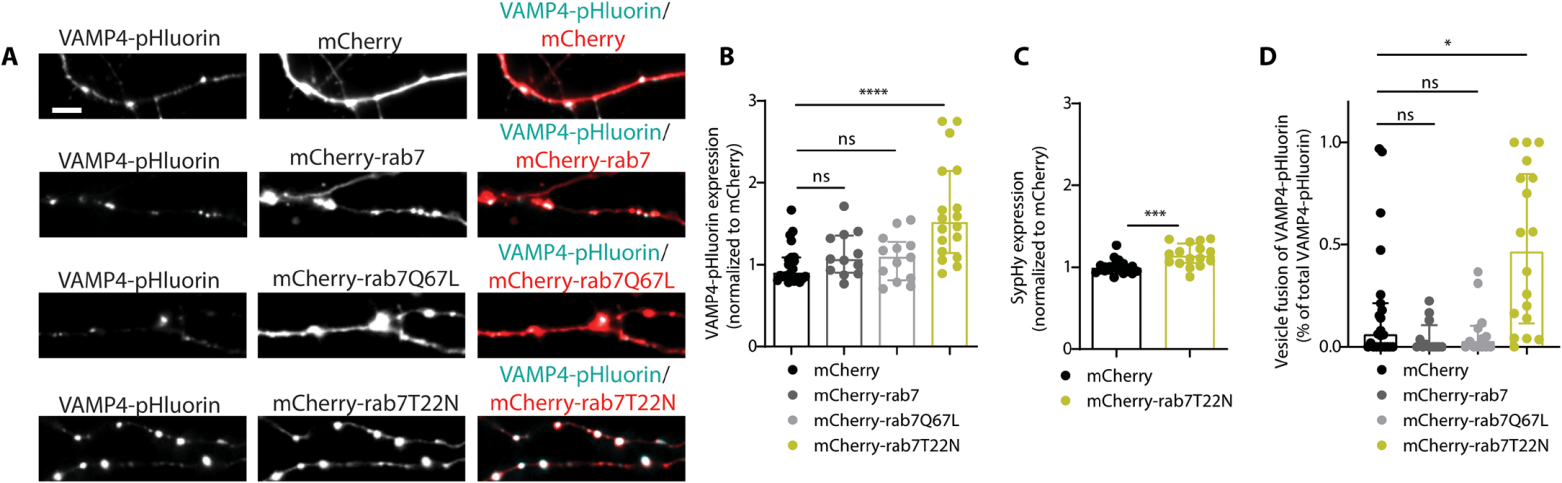
Endolysosomal sorting of VAMP4 regulates its abundance in the SV pool. (**A** and **B**) Hippocampal neurons cotransfected with VAMP4-pHluorin and either wild-type (WT), constitutively active (Q67L), or dominant-negative (T22N) mCherry-rab7. Scale bar, 5 μm. Representative images (A) and bar graph (B) display synaptic VAMP4-pHluorin expression. *n* = 23 (mCherry), *n* = 12 (mCherry-rab7), *n* = 13 (Q67L), and *n* = 18 (T22N) coverslips from four independent preparations, ****P < 0.001, Kruskal-Wallis test with Dunn’s multiple comparisons test. (**C**) Effect of T22N mCherry-rab7 overexpression on sypHy synaptic expression. *n* = 21 (mCherry) or *n* = 16 (T22N mCherry-rab7) coverslips from four independent preparations, ***P < 0.0002, Mann-Whitney test. (**D**) Fusion events reported by VAMP4-pHluorin upon stimulation with 400 APs at 40 Hz under the conditions shown in (A) and (B), *n* = 23 (mCherry), *n* = 12 (mCherry-rab7), *n* = 13 (Q67L), and *n* = 18 (T22N) coverslips from four independent preparations, *P = 0.0129, Kruskal-Wallis test with Dunn’s multiple comparisons test.

To determine the importance of endolysosomal trafficking of VAMP4 in its axonal turnover, we expressed either constitutively active (Q67L) or dominant-negative (T22N) mutants of the small GTPase rab7 and evaluated their effects on the synaptic expression of VAMP4. Q67L rab7 cannot hydrolyze guanosine 5′-triphosphate (GTP), but the effect of its overexpression on organelle trafficking in axons is controversial (*29*, *30*). In contrast, T22N rab7 has a reduced affinity for GTP, and its overexpression significantly dysregulates axonal endosome transport and signaling (*30*–*32*). Expression of either wild-type mCherry-rab7 or the Q67L mutant had no impact on axonal expression of VAMP4-pHluorin (Fig. 4, A and B). In contrast, expression of T22N mCherry-rab7 significantly increased the synaptic expression of VAMP4-pHluorin by approximately twofold (Fig. 4, A and B). The synaptic expression of sypHy was also increased in neurons expressing T22N mCherry-rab7, however to a much smaller extent (Fig. 4C). This implies that VAMP4 is disproportionately trafficked away from nerve terminals via the endolysosomal system in axons. Inhibition of endolysosomal trafficking not only increased the abundance of VAMP4-pHluorin in nerve terminals but also stimulated its incorporation in the recycling SV pool (Fig. 4D). This was indicated by the increased fusion of VAMP4-pHluorin in response to stimulation (40 Hz, 10 s) in T22N rab7–expressing neurons (Fig. 4D). Thus, VAMP4 provides a molecular link between the endolysosomal and SV recycling machineries.

**Fig. 5:**
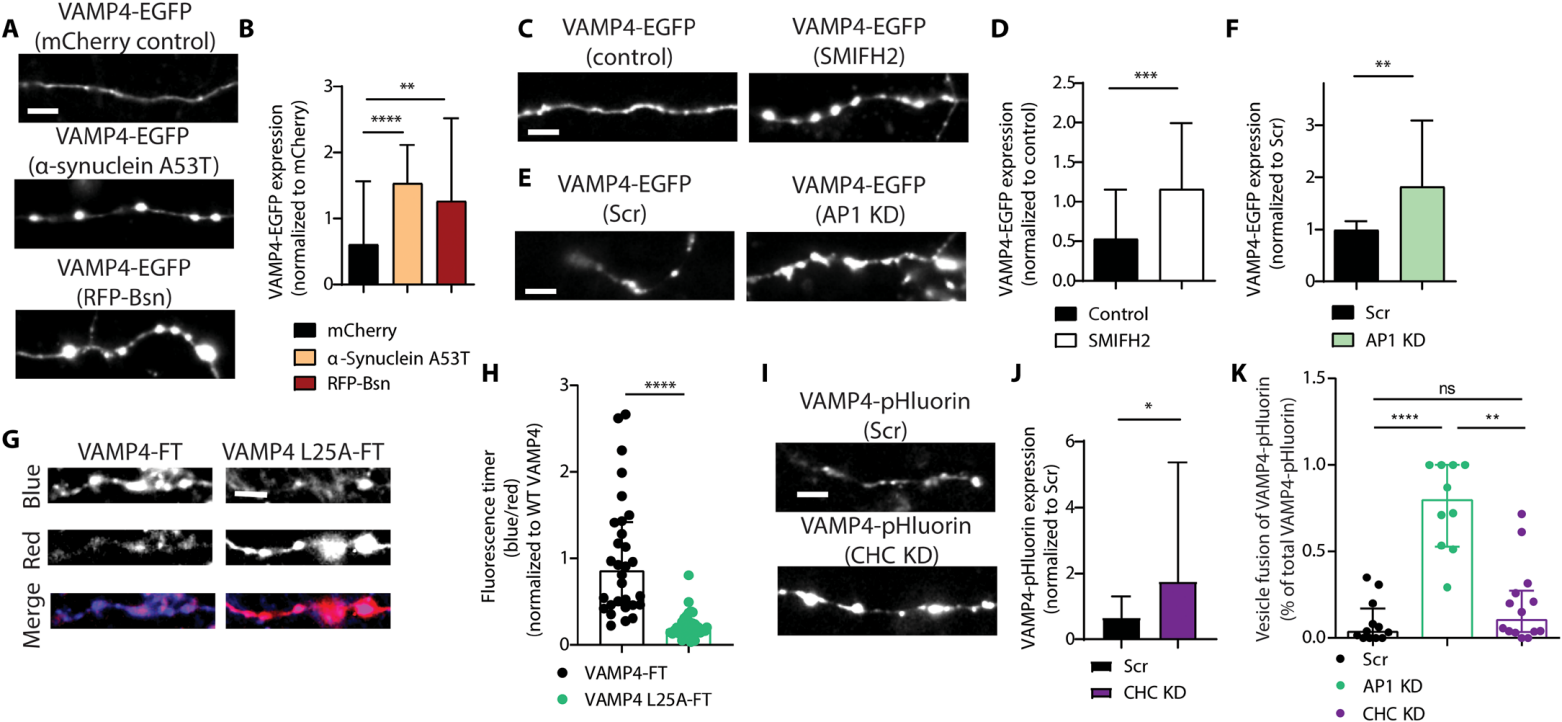
AP1-mediated sorting of VAMP4 to endolysosomes regulates its abundance in the SV pool. (**A**, **C**, and **E**) Representative images display hippocampal neurons transfected with VAMP4-EGFP and mCherry, α-synuclein A53T, or red fluorescent protein (RFP)–bassoon (Bsn) (A), VAMP4-EGFP–transfected neurons with and without 30 μM SMIFH2 (C) and VAMP4-EGFP cotransfected with either AP1 shRNA (AP1 KD) or a scrambled (Scr) control (E). Scale bars, 5 μm. (**B**, **D**, and **F**) Synaptic VAMP4 expression under all conditions described in (A), (C) and (E). (B) *n* = 40 (mCherry), *n* = 51 (α-synuclein A53T), and *n* = 53 (bassoon) cells from three independent preparations, ****P < 0.001 and **P = 0.0038, Kruskal-Wallis test with Dunn’s multiple comparisons test (*P* values adjusted). (D) *n* = 47 (control) and *n* = 61 (SMIFH) cells from three independent preparations (F), *n* = 18 coverslips (scrambled and AP1 KD) from four independent preparations, ***P = 0.0009 and **P = 0.0085, Mann-Whitney test. (**G** and **H**) Hippocampal neurons were transfected with either wild-type or VAMP4 L25A–FT. (G) Representative images display fluorescence in the blue (new protein), red (old protein), or merged channel. Scale bar, 5 μm. (H) Blue/red ratio [*n* = 30 (wild-type) and *n* = 39 (L25A) cells from four independent preparations, ****P < 0.001, Mann-Whitney test]. (**I** to **K**) Hippocampal neurons cotransfected with VAMP4-pHluorin and or shRNAs against either AP1 or CHC (or scrambled). Representative images (I) (scale bar, 5 μm) and quantification of VAMP4-pHluorin expression (J) in either scrambled or CHC KD synapses [*n* = 12 (scrambled), *n* = 14 (CHC KD) coverslips from three independent preparations, *P = 0.011, Mann-Whitney test]. (K) VAMP4-pHluorin fusion events [*n* = 12 (scrambled), *n* = 10 (AP1 KD), and *n* = 14 (CHC KD) coverslips from three independent preparations, ****P < 0.0001 and **P < 0.01, Kruskal-Wallis test with Dunn’s multiple comparisons test].

The endolysosomal pathway plays a central role in synaptic senescence and neurodegeneration, a common feature of which is the accumulation of insoluble protein species and protein aggregates that lead to lysosomal dysfunction (*33*). Therefore, we next determined whether inhibition of the endolysosomal degradation pathway with cytotoxic proteins perturbed the presynaptic turnover of VAMP4. To achieve this, we overexpressed two different aggregation-prone proteins known to be cleared by the lysosome (Fig. 5A). These were (i) a pathogenic mutant of α-synuclein (A53T) proposed to act via inhibition of lysosomal degradation (*34*, *35*) and (ii) one of the main components of the presynaptic cytomatrix, Bassoon, which forms large aggregates in neuronal cell bodies and drives neurodegeneration in multiple sclerosis (fig. S4A) (*36*). Both interventions significantly increased the synaptic expression of VAMP4-EGFP (Fig. 5, A and B), supporting the notion that burdening the endolysosomal degradation system results in increased synaptic expression of VAMP4.

At the synapse, VAMP4 is trafficked through ADBE during synaptic activity (*5*). Therefore, we next determined whether its endocytic trafficking through bulk endosomes is required for targeting to endolysosomes. ADBE is triggered by intrinsic neuronal network activity in our neuronal cultures, as indicated by the robust uptake of the fluid phase marker tetramethylrhodamine (TMR)–dextran (40 kDa) (fig. S4B). We next determined whether two independent interventions that disrupted ADBE at different stages of the process also increased VAMP4 levels at the presynapse. These were the treatment with the formin inhibitor small molecule inhibitor of formin homology 2 domain (SMIFH2), which blocks the formation of bulk endosomes (*37*), or short hairpin RNA (shRNA)–mediated depletion of AP1, which impairs the budding of vesicles from presynaptic endosomes (*38*, *39*) (fig. S4, C and D). In both instances, inhibition of ADBE resulted in robust accumulation of VAMP4 at the presynapse (Fig. 5, C to F).

To determine whether disrupting AP1 binding also affected the synaptic turnover of VAMP4, we examined an AP1 interaction–deficient VAMP4 mutant [VAMP4 L25A (*12*)] tagged with FT. VAMP4 L25A–FT exhibited a markedly reduced blue-to-red ratio in nerve terminals, when compared to wild-type VAMP4-FT (Fig. 5, G and H). This suggests that in addition to regulating its presynaptic expression, AP1 binding facilitates the synaptic turnover of VAMP4. We next determined whether the presynaptic accumulation of VAMP4 in AP1-depleted neurons affected its activity-dependent (40 Hz, 10 s) trafficking. AP1 KD (knockdown) resulted in a phenotype similar to that observed in T22N rab7–expressing neurons, namely, a marked increase in the number of VAMP4-pHluorin fusion events during high-frequency stimulation (Figs. 4D and 5K). This reveals that in the absence of AP1, VAMP4 accumulates in the recycling SV pool.

As a heterotetrameric adaptor protein, AP1 loads cargo into nascent vesicles by interacting with the clathrin lattice. Therefore, we next determined whether depletion of clathrin heavy chain (CHC; fig. S4, E and F) affected the expression and activity-dependent trafficking of VAMP4-pHluorin in a manner similar to AP1 KD. In agreement, CHC KD neurons exhibited increased accumulation of VAMP4 in nerve terminals (Fig. 5, I and J). However, this was not accompanied by an increase in evoked VAMP4-pHluorin fusion events (Fig. 5K). These data suggest that AP1 and clathrin play partly overlapping but not identical roles in the presynaptic sorting of VAMP4, with AP1 specifically sorting VAMP4 to endolysosomes, and clathrin potentially required for sorting of VAMP4 to both endolysosomes and SVs.

Together, these data indicate that the presynaptic abundance and activity-dependent trafficking of VAMP4 constitute a tunable mechanism that reflects the functionality status of both SV recycling and the endolysosomal degradation machinery. These results also uncover a previously unidentified role for the endocytic adaptor AP1 in targeting of SV cargo to endolysosomes during SV recycling.

### VAMP4 KO synapses display a reduction in endolysosomal molecules but no differences in the SV proteome

We have demonstrated that VAMP4 copy number in the SV pool limits SV fusogenicity and that its presynaptic expression is determined by its clearance via ADBE and targeting to endolysosomes. To determine how these events are perturbed in the absence of VAMP4, we generated a *Vamp4* KO mouse line using CRISPR-Cas9–mediated gene editing (fig. S5A). Deletion of the *Vamp4* gene did not disrupt the Mendelian distribution of offspring or cause premature mortality. However, male KO mice were infertile due to a defect in spermatogenesis, consistent with an essential role for VAMP4 in sperm head development and acrosome vesicle fusion (*40*).

**Fig. 6:**
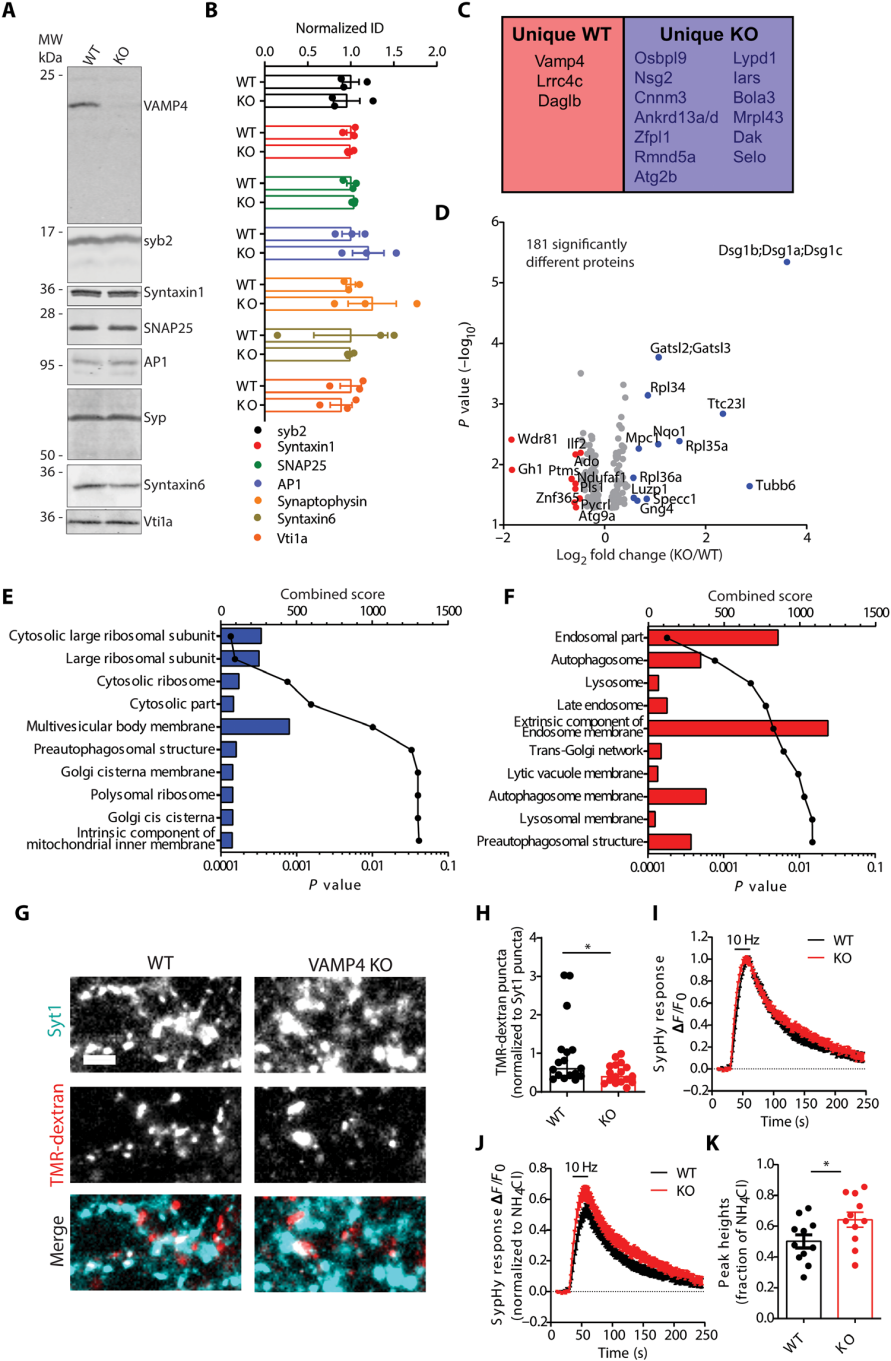
VAMP4 KO synapses display a reduction in endolysosomal molecules and increased SV fusion. (**A** and **B**) Wild-type and VAMP4 KO brain lysates were immunoblotted for synaptic and endosomal proteins (all *n* = 3 animals, *t* test with Benjamini, Krieger, and Yekutieli correction for false discovery rate). MW, molecular weight. (**C** to **F**) MS analysis of synaptosomes from wild-type and VAMP4 KO brains (five animals for both wild-type and VAMP4 KO). (C) Unique proteins. (D) Volcano plot of significantly different proteins. Refer to data file S1 for full protein names in C and D. (E and F) GO cellular compartment analysis of increased/unique (E) and decreased/absent (F) proteins in VAMP4 KO synaptosomes. (**G** and **H**) Wild-type and VAMP4 KO cultures incubated with fluorescent-conjugated antibodies to synaptotagmin 1 (Syt1) were stimulated with 400 APs (40 Hz) and 50 μM TMR-dextran. (G) Representative images (Syt1, TMR-dextran, and merged). Scale bar, 5 μm. (H) TMR-dextran puncta per field of view normalized to Syt1 puncta. *n* = 17 coverslips from three independent preparations (both), *P = 0.0238, Mann-Whitney test. (**I** and **J**) Average time course of the sypHy response normalized to either the stimulation peak (I) or total SV pool [revealed by NH_4_Cl application (J)]. Stimulation indicated by bar. (**K**) Peak sypHy response as a fraction of the total SV pool. *n* = 11 coverslips from three independent preparations (both), *P = 0.043, unpaired *t* test.

VAMP4 protein expression was abolished in the brains of KO animals as demonstrated by both immunoblotting and IHC (Fig. 6A and fig. S5B). IHC in brain sections revealed no obvious differences between wild-type and VAMP4 KO mice in their cytoarchitecture (fig. S5C). Furthermore, no significant differences in the protein levels of potentially functionally redundant SNARE, presynaptic, and Golgi proteins were detected in either brain homogenates or isolated nerve terminals (synaptosomes) derived from VAMP4 KO mice (Fig. 6, A and B, and fig. S5, D and E). These results indicate that deletion of the *Vamp4* gene has no general adverse effect on synaptic architecture, with no obvious compensatory alterations in protein expression.

To determine whether the absence of VAMP4 disrupted specific molecular events within the presynapse, we next performed a proteomic screen of synaptosomes prepared from brains of either wild-type or VAMP4 KO mice. This profiling identified 3159 proteins and a small cohort that significantly changed expression between wild-type and VAMP4 KO synaptosomes (Fig. 6, C and D, and data file S1). In addition, specific proteins were uniquely expressed in either wild-type or VAMP4 KO synaptosomes (Fig. 6C). To determine how the absence of VAMP4 affected specific cellular processes, we performed gene ontology (GO) analysis on uniquely expressed proteins, in addition to proteins that displayed a fold change of >1.5 or <0.72 in VAMP4 KO synaptosomes and were significantly different (*P* < 0.05) from wild-type. Increased expression of proteins linked to ribosomal function was observed in VAMP4 KO synaptosomes (Fig. 6E). In contrast, decreased or absent proteins in VAMP4 KO synaptosomes were clustered around compartments such as endosomes, lysosomes, and autophagosomes (Fig. 6F). This agrees well with the selective trafficking of VAMP4 via the endolysosomal system and suggests a role in the regulation of the flux of specific molecules through this pathway. The absence of any significant accumulation of SV cargos in VAMP4 KO nerve terminals strongly indicates that the absence of VAMP4 does not have any global impact on the SV proteome. Instead, it suggests that the copy number of VAMP4 in the SV pool, as well as its selective removal through the endolysosomal system, is a precise and finely adjustable mechanism to control Pr in response to changes in neuronal activity and proteostasis.

### VAMP4 KO neurons display a specific augmentation of SV fusion

We next determined whether deletion of the *Vamp4* gene affected the structural and functional integrity of synapses. Ultrastructural analyses of wild-type and VAMP4 KO nerve terminals in culture revealed no differences in either synaptic morphology or SV number (fig. S5, F to H). Furthermore, immunocytochemistry (ICC) against specific synaptic proteins revealed no obvious differences (fig. S5, I and J).

We next examined whether SV recycling was affected by ablation of VAMP4 expression. First, ADBE was monitored by uptake of TMR-dextran during a train of high-frequency APs (40 Hz, 10 s). The number of nerve terminals performing ADBE was significantly reduced in VAMP4 KO neurons (Fig. 6, G and H). However, the number of bulk endosomes in individual synaptic terminals undergoing ADBE was not affected, as revealed by an ultrastructural analysis of the uptake of horseradish peroxidase (HRP) in response to strong stimulation (fig. S5, K to M). We next determined whether the kinetics of SV recycling was affected in VAMP4 KO neurons using sypHy. SypHy can reveal the speed of SV endocytosis by monitoring the time course of its fluorescence quenching after stimulation, since the rapid acidification of SVs renders endocytosis as the rate-limiting step (*41*, *42*). The kinetics of sypHy quenching was unaltered in VAMP4 KO neurons after challenge with an AP train (10 Hz, 30 s), suggesting no obvious role for VAMP4 in SV endocytosis (Fig. 6I). The evoked amplitude of the sypHy response (peak height) was significantly higher in VAMP4 KO neurons (Fig. 6, J and K). This implies that SV fusion is enhanced in KO neurons, further supporting the notion that VAMP4 acts as a negative regulator of SV exocytosis.

### Hippocampal VAMP4 KO circuits display increased Pr and an inability to sustain presynaptic facilitation

**Fig. 7:**
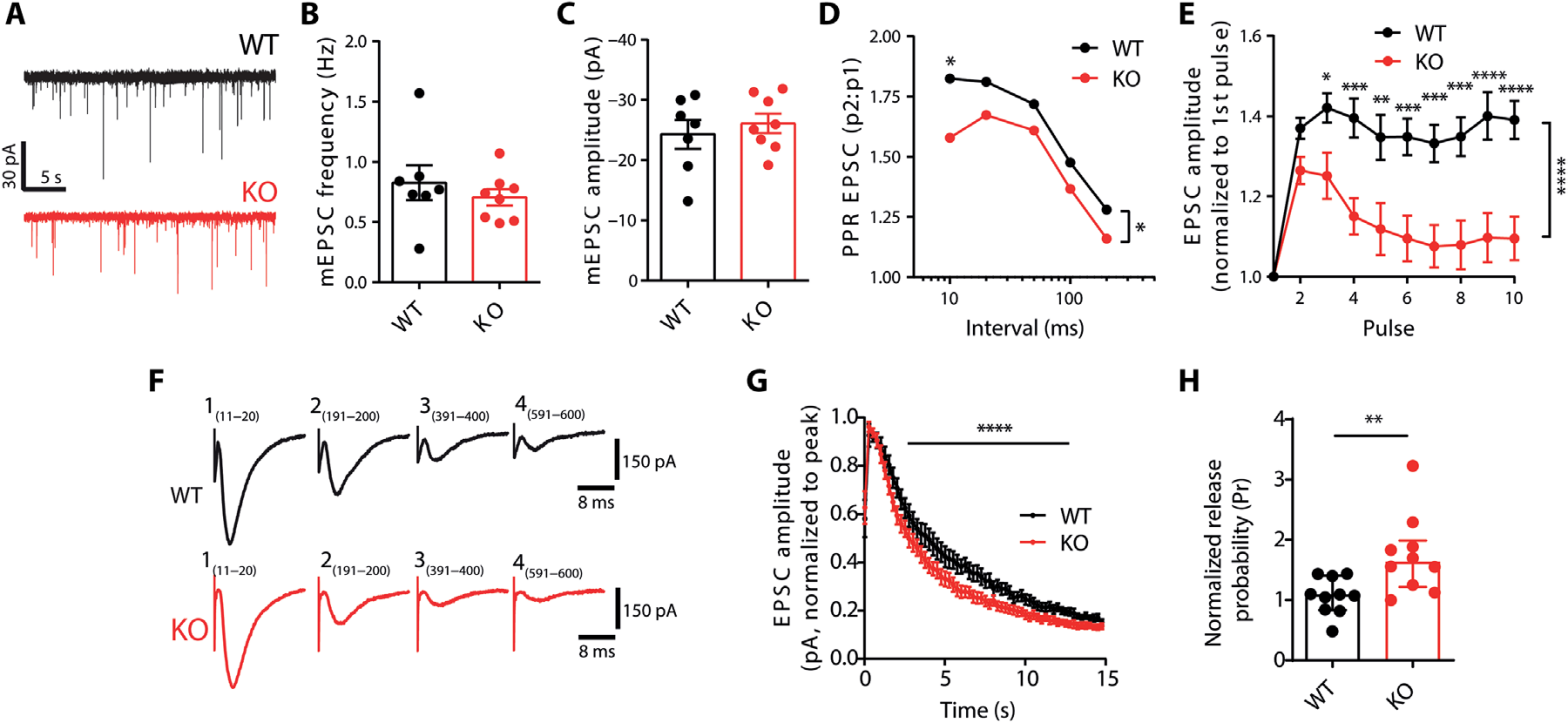
Increased Pr in hippocampal VAMP4 KO circuits. Neurotransmission at CA3-CA1 synapses was monitored using whole-cell patch-clamp recording in acute hippocampal slices from wild-type and VAMP4 KO mice. (**A**) Example mEPSC events. Frequency (**B**) and amplitude (**C**) of mEPSC events. *n* = 7 (wild-type) or *n* = 8 (KO) slices from four animals, Mann-Whitney test. (**D**) Paired-pulse ratio (PPR) of EPSCs as a function of the interstimulus interval (10 to 200 ms). *n* = 17 (wild-type) or *n* = 15 (KO) slices from either 10 or 9 animals, *P < 0.05, **P < 0.01, ***P < 0.001, ****P < 0.0001, pulse number, two-way ANOVA with Fisher’s least significant difference, *P = 0.027, two-way ANOVA for genotype. (**E**) Evoked EPSC amplitude for slices stimulated with a 10-AP train (20 Hz, normalized to the first pulse). *n* = 10 (wild-type) or *n* = 9 (KO) slices from either seven or six animals, ****P < 0.0001, two-way ANOVA. (**F** and **G**) Slices were stimulated with 600 APs (40 Hz). Example EPSC amplitudes (F), average EPSC amplitude (normalized to peak response) (G). *n* = 10 (both) slices from either 6 or 8 animals, ****P < 0.0001, two-way ANOVA. (**H**) Pr of the CA3-CA1 synapse in wild-type and VAMP4 KO, calculated by dividing the amplitude of the first evoked EPSC by the effective RRP size. *n* = 10 (both) slices from either six or eight animals, **P = 0.039, Mann-Whitney test.

To determine whether the observed enhancement of SV exocytosis in hippocampal cultures translated into defects in neurotransmission in intact brain circuits, we examined the effect of the absence of VAMP4 at cornu ammonis (CA)3-CA1 hippocampal synapses using whole-cell patch-clamp recordings. No differences in either the intrinsic properties (table S1) or the frequency or amplitude of miniature excitatory postsynaptic currents (mEPSCs) were detected between wild-type and VAMP4 KO synapses (Fig. 7, A to C), suggesting that the absence of VAMP4 does not significantly affect the postsynaptic neuron or AP-independent neurotransmission.

We next determined the effect of VAMP4 KO on evoked neurotransmission. A comparison of wild-type and VAMP4 KO evoked EPSCs elicited by delivering stimuli at a range of intensities (25, 50, 75, and 100 μA) revealed an increase in EPSC amplitudes in VAMP4 KO synapses across the entire stimulus range (fig. S6A). When CA3 axons were stimulated with pairs of pulses at a range of interstimulus intervals, a significant decrease in the paired-pulse EPSC ratio was observed in VAMP4 KO slices when compared to wild type (Fig. 7D). Furthermore, a pronounced increase in the decay of synaptic facilitation was detected in VAMP4 KO slices during both short (10 APs at 20 Hz; Fig. 7E) and long (600 APs at 40 Hz; Fig. 7, F and G) trains of AP stimulation at high frequency. This indicates that VAMP4 contributes to the tuning of the synaptic responses to changing patterns of activity and is specifically required for synaptic facilitation at the Schaffer collateral–CA1 synapse. The presynaptic origin of this increased short-term depression of neurotransmission was confirmed in cultured hippocampal neurons using the genetically encoded glutamate sensor, iGluSnFR (*43*). When VAMP4 KO neurons were challenged with short AP bursts (10 APs at 20 Hz; as in Fig. 7E), a significant depression of the iGluSnFR response in axons was observed when compared to wild type (fig. S6, B and C).

A reduction in the paired-pulse ratio and enhanced synaptic rundown during stimulation at high frequency typically reflect elevated initial Pr (*44*). To obtain a precise measure of Pr and assess whether it was altered in VAMP KO circuits, the amplitude of the first evoked EPSC was divided by the effective RRP size, estimated from an AP train at 40 Hz (fig. S6D) (*45*). Neither RRP size nor its replenishment during the stimulus train was significantly different between wild type and KO (fig. S6, E and F); however, Pr was significantly higher in VAMP4 KO slices (Fig. 7H). We conclude that the CA3-CA1 circuit within the VAMP4 KO hippocampus displays increased Pr, resulting in significant alterations in the time course of synaptic facilitation.

**Fig. 8:**
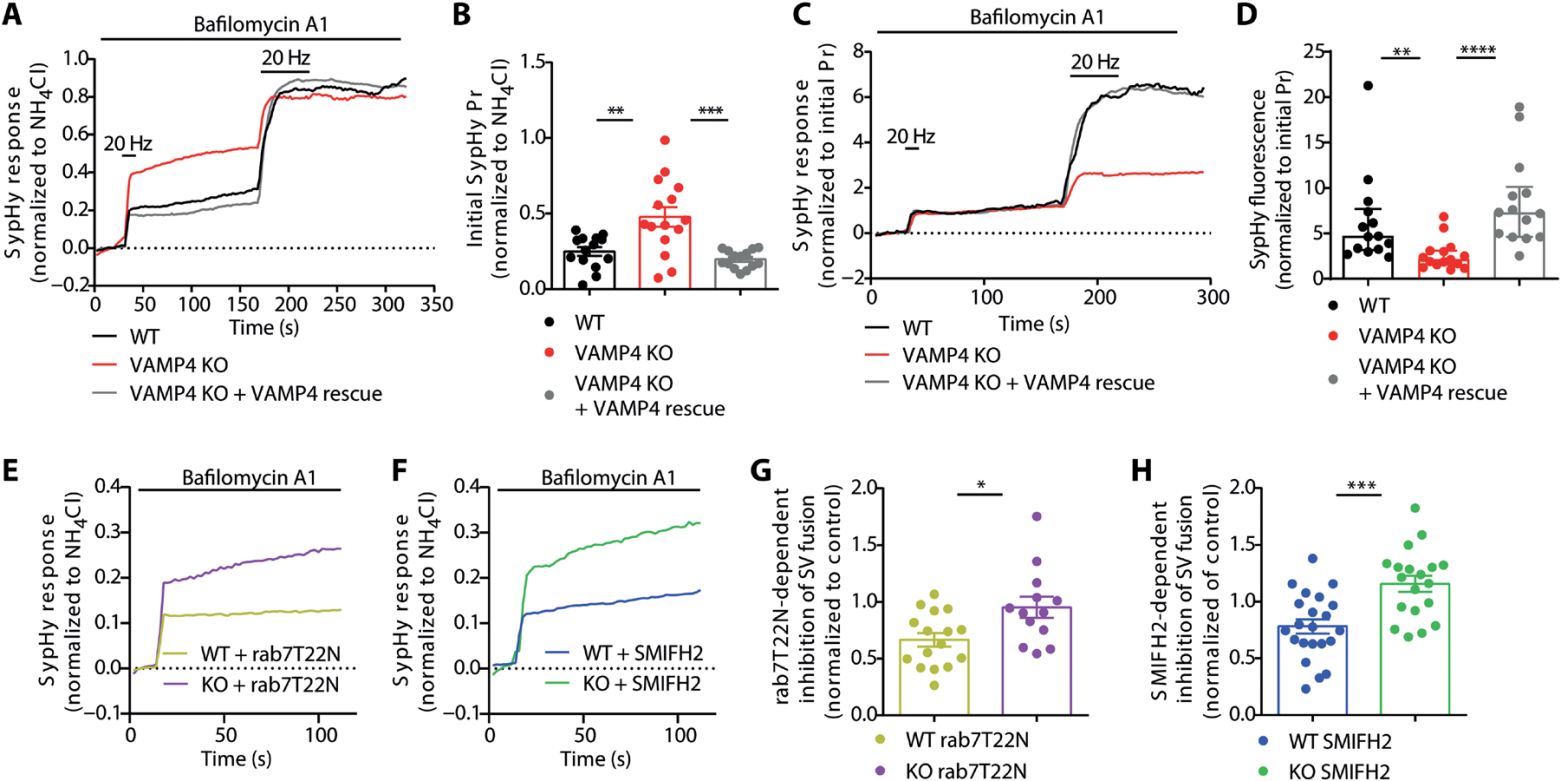
VAMP4 conveys endolysosomal dysfunction into inhibition of SV fusion. (**A** to **D**) Primary cultures of hippocampal neurons transfected with sypHy or sypHy + VAMP4-FT (VAMP4 rescue) were incubated with 1 μM bafilomycin A1 and stimulated with two 20-Hz AP trains (2 and 45 s, indicated by bar), followed by NH_4_Cl solution. Average traces (A) and initial evoked sypHy response normalized to the total SV pool (B). Average traces (C) and sypHy response normalized to the initial response (Pr) (D). *n* = 14 (wild-type, KO/rescue) or *n* = 15 (KO) coverslips from eight independent preparations, (B) **P = 0.0015 and ***P = 0.0001, one-way ANOVA with Tukey’s multiple comparisons test, and (D) **P = 0.0077 and ****P < 0.0001, Kruskal-Wallis test with Dunn’s multiple comparisons test. (**E** to **H**) Identical experiments to those in (A) to (D) were performed in neurons expressing either mCherry-rab7T22N or treated with 30 μM SMIFH2. (E and F) Average traces. (G and H) Inhibition of the sypHy response by either mCherry-rab7T22N (G) or SMIFH2 (H) normalized to wild-type or VAMP4 KO controls. *n* = 16 (wild-type/T22N), *n* = 13 (KO/T22N), *n* = 22 (wild-type/SMIFH2), and *n* = 19 (KO/SMIFH2) coverslips from either four (G) or three (H) independent preparations, *P = 0.012 and ***P = 0.0003, unpaired *t* test.

To confirm the specificity of the observed effect on Pr in a more tractable system, we quantified the extent of SV fusion in primary hippocampal cultures using sypHy imaging. SV exocytosis was elicited by sequential stimulation trains (40 and 900 APs, both delivered at 20 Hz) (Fig. 8A). To observe SV fusion events without contamination from SV endocytosis, we performed the experiment in the presence of bafilomycin A1, a V-type adenosine triphosphatase inhibitor that blocks SV reacidification, allowing the integration of fluorescence amplitudes of all release events (*46*). These experimental settings are commonly used to measure the sizes of RRP and total recycling pool (TRP), which typically scale together (*47*). In VAMP4 KO neurons, the fluorescence amplitudes evoked by a 40-AP train were markedly increased when compared to wild-type neurons (Fig. 8, A and B). This was not due to a change in the TRP, since the sypHy response after the subsequent train of 900 APs was equal between VAMP4 KO and wild type (Fig. 8A). When we normalized the sypHy response evoked by the second train of 900 APs to the initial PR, a robust depression of SV fusion was revealed in VAMP4 KO neurons, consistent with an increase in Pr (Fig. 8, C and D). Both elevated Pr and augmented release depression were fully restored by overexpression of wild-type VAMP4 in KO neurons (Fig. 8, A to D). This suggests that the absence of VAMP4 increases the fusion competence of SVs in the RRP without affecting the total number of recycling SVs. Together, these findings support a model according to which VAMP4 plays a critical role in the control of short-term synaptic facilitation by modulating Pr during trains of APs.

### VAMP4 translates endolysosomal dysfunction into altered presynaptic release properties

The endocytic retrieval of VAMP4 at presynaptic terminals through ADBE and its subsequent sorting to endolysosomes would be an elegant mechanism to calibrate SV fusogenicity to both prior neuronal activity and the demands imposed on the quality control mechanisms that regulate cellular proteostasis. If true, then the inhibition of ADBE-mediated trafficking of VAMP4 to endolysosomes should have significant effects on SV fusion. To address this question, we measured the amount of SV fusion in both wild-type and VAMP4 KO neurons upon blockage of either axonal endolysosomal trafficking or ADBE. SV fusion was assessed as before by monitoring sypHy responses evoked by stimulation with 40 APs delivered at 20 Hz (fig. S7A).

We examined the impact of disrupting endolysosomal trafficking or ADBE via the expression of dominant-negative T22N rab7 or treatment with the formin inhibitor SMIFH2, respectively. Both of these interventions caused extensive synaptic accumulation of VAMP4 (Figs. 4, A and B, and 5D) and resulted in marked inhibition of SV fusion in wild-type neurons (Fig. 8, E and F, and fig. S7, B and D). The effects of disrupting endosomal SV recycling and endolysosomal trafficking on SV fusion were largely occluded in VAMP4 KO neurons (Fig. 8, E and F, and fig. S7, C and E). This was emphasized when the extent of inhibition of the sypHy response by T22N mCherry-rab7 or SMIFH2 was assessed. Wild-type neurons displayed a robust reduction, whereas VAMP4 KO neurons displayed almost none (Fig. 8, G and H, and fig. S7, C and E). Therefore, the presence of VAMP4 was essential to translate inhibition of endolysosomal function into a reduction in Pr.

## DISCUSSION

In this study, we present evidence demonstrating that VAMP4 is a negative regulator of Pr and modulation of its abundance in SVs bestows differences in their fusogenicity. We demonstrate two intricately linked mechanisms that regulate VAMP4 abundance in nerve terminals. Trafficking via ADBE and the endolysosomal system are both essential for maintaining a low steady-state level of VAMP4 at the presynapse. Acting in concert, these two trafficking routes provide a mechanism for activity- and proteostasis-dependent adjustments in synaptic efficacy.

### VAMP4 is a negative regulator of Pr

We present multiple stands of evidence that VAMP4 limits Pr in both neuronal cultures and acute hippocampal slices. For example, VAMP4 expression at individual synapses negatively correlates with the amplitude of the presynaptic responses. We also demonstrate changes in both paired-pulse plasticity and the decay of synaptic facilitation during high-frequency stimulation in VAMP4 KO slices, consistent with Pr being the main determinant of presynaptic short-term plasticity (*44*, *48*). Our results reveal that the control of Pr by VAMP4 is not via modulation of the number of fusion-competent SVs or via the size or replenishment rate of RRP but instead governed by differences in Pr per SV. Synaptic facilitation is a form of synaptic plasticity widely used in the CNS, which enhances neurotransmitter release under conditions in which the pool of releasable SVs decreases (*49*). By definition, repeated bursts of high-frequency synaptic activation should result in depletion of RRP and short-term depression. However, at many synapses, Pr is instead increased in an activity-dependent manner, an effect attributed to the residual calcium signal that becomes available during high-frequency stimulation (*49*). Our results reveal the possibility that the activity-dependent removal of negative regulators of Pr, such as VAMP4, from the SV pool may play an equally important role in counteracting synaptic depression during high-frequency neurotransmission.

VAMP4 plays a role in two other calcium-dependent aspects of neurotransmission, asynchronous release (*14*, *50*) and the frequency of calcium-dependent mEPSCs (*50*). Asynchronous release performs important roles in certain forms of plasticity at specialized synapses but is not likely to be involved in the fast neuronal processing in the CNS, which instead is mediated by synchronous release (*15*, *16*). Our work uncovers a global effect of VAMP4 on neurotransmitter release, in agreement with its ubiquitous expression. Additional roles for VAMP4 in other forms of release in specialized neuronal populations may still exist but will complement its universal effect on Pr. Furthermore, the previously observed reduction in asynchronous release (*14*) could be an indirect consequence of the increased Pr at VAMP4-depleted synapses, since synchronous and asynchronous releases are inversely correlated, due to sharing the same pool of releasable SVs (*51*, *52*). We do not observe any change in mEPSC frequency in VAMP4 KO slices, in contrast to previous observations (*50*). At present, we cannot provide a mechanistic explanation for these differences; however, it may reflect the experimental systems in which they were performed (shRNA knockdown in primary culture versus genomic deletion in intact neuronal circuits).

Our results suggest that the presence of VAMP4 on SVs does not act as a binary switch for SV fusion but instead has a modulatory effect. This is because SVs that contain VAMP4 can still undergo synchronous activity-dependent fusion. The high degree of similarity between the SNARE motifs of syb2 and VAMP4 raises the possibility that VAMP4 engages in interactions with the canonical Q-SNAREs (fig. S2A). However, our FRET-FLIM data strongly suggest that this interaction is ineffective during synaptic activity. Hence, the inhibitory effect of the synaptic accumulation of VAMP4 on SV fusion may be a direct consequence of its ineffective pairing with the canonical Q-SNAREs. This coupling would result in the buffering of the Q-SNAREs in structurally unstable and less fusion-competent complexes and thus preclude them from interacting with their cognate R-SNARE partners (such as syb2). We therefore speculate that the relative abundance of VAMP4 in relation to positive (such as syb2) or negative [such as amisyn (*53*)] regulators of exocytosis determines the net fusion competence of an individual SV. Therefore, the mechanisms modulating the presynaptic levels of VAMP4 provide an attractive lever for the fine-tuning of synaptic strength to the specific requirements of neuronal circuits.

### ADBE and endolysosomal trafficking regulate the abundance of VAMP4 in the SV pool

**Fig. 9:**
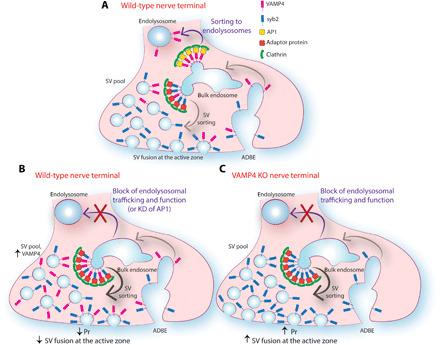
VAMP4 targeting to endolysosomes controls Pr. (**A**) Strong synaptic stimulation triggers ADBE, which recycles SV membranes and proteins, such as syb2 and VAMP4, following SV fusion. ADBE forms transient bulk endosomes from which vesicles bud to (i) refill the recycling SV pool (ii) or fuse with (mature to) endolysosomes for long-range trafficking and degradation. Proteins are sorted at bulk endosomes to either endolysosomes or SVs by different mechanisms. VAMP4 is predominantly sorted to endolysosomes via an AP1-dependent mechanism, whereas syb2 and, to a lesser extent, VAMP4 are sorted into SVs by different adaptor proteins. Both sorting pathways require clathrin for their function. (**B**) In wild-type nerve terminals, inhibition of either endolysosomal trafficking or AP1 function abolishes VAMP4 sorting to endolysosomes and results in its increased sorting in SVs. VAMP4 accumulation in the SV pool reduces SV fusion capacity and Pr, although increased numbers of VAMP4 molecules visit the surface during stimulation. (**C**) In VAMP4 KO terminals, loss of VAMP4 causes an increase in Pr under basal conditions due to loss of inhibitory control. In addition, blocking of endolysosomal trafficking and function does not affect Pr, highlighting the essential role of VAMP4 in converting endolysosomal dysfunction into changes of presynaptic release properties.

VAMP4 is selectively retrieved via ADBE, and its enrichment in presynaptic bulk endosomes has been confirmed at the ultrastructural level (*5*). Although a small fraction (25%) of VAMP4 participates in SV recycling at any given time, its accumulation at silent synapses indicates that both synaptic activity and ADBE are required for its clearance from synaptic terminals. We also demonstrated that ADBE is essential for the presynaptic clearance of VAMP4 using two independent interventions that limit ADBE: inhibition of formin (*37*) and depletion of AP1 (*38*, *39*). The impact of ADBE inhibition on presynaptic VAMP4 levels suggests that most of the bulk endosome–derived VAMP4 vesicles are cleared via axonal retrograde trafficking. However, some VAMP4 vesicles recycle and reincorporate in the SV pool, agreeing with previous studies showing that ADBE provides a cohort of SVs to replenish the SV reserve pool (*39*, *54*). Thus, bulk endosomes may be a key presynaptic sorting station, which generates both functional reserve pool SVs and vesicles destined for degradation (Fig. 9A). Furthermore, the accumulation of VAMP4 in the SV pool in response to depletion of AP1 suggests a new synaptic role for this endocytic adaptor in targeting of SV cargo to endolysosomes. In addition to AP1, the cytoplasmic domain of VAMP4 contains motifs for interaction with AP180, CALM (clathrin assembly lymphoid leukemia protein), and PACS-1 (phosphofurin acidic cluster sorting protein-1) (*12*, *55*, *56*). Therefore, it is possible that an interplay between different endocytic adaptors controls the distribution of VAMP4 between the SV pool and endolysosomes, with AP1 being the master regulator.

Our findings indicate that in addition to being a sorting intermediate in SV biogenesis, bulk endosomes integrate SV recycling with the intracellular trafficking machinery that maintains protein homeostasis: the endolysosomal system. The removal of VAMP4 from the SV pool through the endolysosomal system was revealed by its unusually high synaptic turnover rate, the retrograde bias of its axonal transport, and cotrafficking with the late endosomal marker rab7. This removal is not only controlled by AP1 binding but may also be facilitated by the VAMP4 polyubiquitination. VAMP4 trafficking via the endolysosomal system was confirmed by our demonstration of VAMP4 accumulation at synaptic terminals during overexpression of a dominant-negative rab7 mutant (*28*) and two cytotoxic proteins (α-synuclein A53T and bassoon), which compromise neuronal proteostasis (*34*–*36*).

Proteomic analysis of VAMP4 KO synaptosomes revealed a selective de-enrichment of proteins linked to endosome, lysosome, and autophagosome function. Therefore, in addition to being a cargo, VAMP4 is required for optimal functioning of these pathways, exerting a bidirectional control of SV recycling and endolysosomal trafficking through dynamic redistribution between the two systems. A different cohort of proteins related to ribosome function was enriched in VAMP4 KO synaptosomes, suggesting that VAMP4, ADBE, and the endolysosomal system are required for efficient synaptic clearance of ribosomes. However, the functional role and precise mechanisms regulating VAMP4-dependent ribophagy at the presynapse remain to be determined.

### Plastic control of neurotransmission via the activity-dependent clearance of VAMP4

Although the precise mechanisms have remained elusive, there is an emerging consensus regarding how endolysosomal trafficking regulates presynaptic efficacy. Thus, excessive SV flux to endolysosomes results in a marked increase in neurotransmitter release (*7*, *57*). Conversely, inhibition of endosomal recycling and trafficking inhibits SV fusion and neurotransmitter release (*7*, *58*). In agreement, we observed a decreased ability of RRP SVs to fuse as a result of overexpression of T22N rab7 mutant. This limit on RRP SV fusion was also observed upon inhibition of ADBE, indicating a close coupling between these two processes. These effects were occluded in VAMP4 KO nerve terminals, confirming that the activity-dependent trafficking of VAMP4 via both ADBE and the endolysosomal system plays an essential role in the regulation of synaptic strength (Fig. 9).

The precise and accurate function of endocytosis pathways is essential for replenishment of the RRP and clearance of the vesicular debris from release sites, both of which enable sustained neurotransmission (*49*, *59*). Our study reveals a new layer of regulation in SV exocytosis-endocytosis coupling. Through the selective, activity-dependent clearance of VAMP4, ADBE regulates the composition of newly generated SVs and their propensity for fusion in the subsequent rounds of neurotransmitter release. This enables feedback control and dynamic adjustment of Pr that is tuned to the history of presynaptic activity while simultaneously reporting the overall functionality of neuronal proteome.

Synaptic proteins accumulate damage during repetitive SV recycling (*20*, *21*). Since endolysosomal dysfunction is central to both synaptic senescence and neurodegeneration (*33*, *60*), VAMP4 may act as a brake on excess excitatory neurotransmission during neuronal pathology. In this scenario, the presynaptic accumulation of VAMP4 may provide a regulatory loop via which an overloaded or dysfunctional endolysosomal degradation system can feed back to the SV fusion machinery to prevent further accretion of damage and preserve synaptic function. In summary, our data support a model in which VAMP4 acts as a molecular rheostat that adjusts Pr to input-specific and cell-wide cues, reflecting the traffic flow from a major SV recycling pathway to the endolysosomal system.

## MATERIALS AND METHODS

### Experimental design

Sample sizes were not predetermined using power analysis, since they were not chosen on the basis of prespecified effect size. Instead, multiple independent experiments were performed using biological replicates. Detailed descriptions of sample sizes and statistical analysis used to test normality and calculate *P* values are given in the “Statistical analysis” section, figure legends, and table S2. Data were collected and analyzed by multiple researchers, blind to the conditions whenever experimentally possible. Our inclusion criteria for the time-series analysis were based on the quality of the collected data: For example, image stacks that displayed no focus drift were included in the analysis. Experiment-specific inclusion criteria are detailed in the respective sections below. No outliers were omitted from our analyses.

The objective of the research was to determine how the interplay between SV recycling and endolysosomal trafficking generates SV heterogeneity and controls neurotransmitter release. We hypothesized that the noncanonical R-SNARE VAMP4 was a molecular lever that reported the status of both SV recycling and proteostasis via regulation of Pr. All experiments were performed in a controlled laboratory setting.

### Animals

Neuronal cells and brain tissue were obtained from *Vamp4* KO, wild-type C57BL/6J mice and wild-type Sprague-Dawley rats of both sexes. Horizon Discovery (St. Louis, MO, USA) generated the *Vamp4* KO mice on a C57BL/6J background. An early stop codon was introduced by a 10–base pair deletion in exon 5 of the *Vamp4* gene (18009-18018 in NC_000067.6) using CRISPR-Cas9 gene editing, resulting in nonsense-mediated mRNA decay and elimination of VAMP4 protein expression. For experiments, heterozygous *Vamp4* males and females were bred to produce wild-type and KO littermate controls.

C57BL/6J mice were sourced from an in-house colony at the University of Edinburgh. Sprague-Dawley rats were sourced from an in-house colony at the Francis Crick Institute. All mouse and rat colonies were housed in standard open-top caging on a 14-hour light/dark cycle (light from 0700 to 2100). Both rat and mouse breeders were fed with RM1 chow, whereas stock rats and mice were maintained on RM3 chow.

All animal work was performed in accordance with the U.K. Animal (Scientific Procedures) Act 1986, under Project and Personal License authority, and was approved by the Animal Welfare and Ethical Review Body at either the University of Edinburgh (Home Office project license to M.A.C.: 70/8878) or the Francis Crick Institute (Home Office project license to S.K.U.: 70/7771). With the exception of perfusion fixation, animals were euthanized by schedule 1 procedures in accordance with U.K. Home Office Guidelines. Adults were euthanized by either cervical dislocation or exposure to rising CO_2_ levels followed by decapitation, whereas embryos were euthanized by decapitation followed by destruction of the brain. Specific mice were subject to perfusion fixation. In this procedure, mice were administered a lethal dose of sodium pentobarbital and transcardially perfused with cold phosphate buffer (PB), followed by cold paraformaldehyde (PFA; 4% in 0.1 M PB; performed under license Home Office project license to M. Nolan, PC198F2A0).

### Materials

The following materials were used in this study: dl-2-amino-5-phosphonopentanoic acid (AP-5; ab120271), 6-cyano-7-nitroquinoxaline2,3-dione (CNQX; ab120044), and SMIFH2 (ab218296) from Abcam; bafilomycin A1 (A8627) from Stratech; Neurobasal (21103-049), l-glutamine (25030-024), B27 supplement (17504-044), penicillin-streptomycin (15140-122), Lipofectamine 2000 (11668-019), NeuroTrace 500/525 green fluorescent Nissl stain (N21480), and ProLong Gold antifade mountant (P36930) from Thermo Fisher Scientific; papain (LK003176) from Worthington; fetal calf serum (S1810-500) from Biosera; FluorSave reagent (345789) from Merck Millipore; poly-d-lysine hydrobromide (P7886), d-laminin (L2020), cytosine β-d-arabinofuranoside (C1768), HRP (P8250), 3,3′-diaminobenzidine (D8001), Durcupan resin (44610), protease inhibitor cocktail (P8849), and picrotoxin (P1675) from Sigma-Aldrich.

### Oligonucleotides

The following oligonucleotides were used to generate plasmids in this study. Mutagenic primers (mutated base underlined) are as follows: α-synuclein A53T, TGGTGCATGGTGTGACAACAGTGGCTGAG (forward) and CTCAGCCACTGTTGTCACACCATGCACCA (reverse); Q67L mCherry-rab7, ATGGGACACAGCAGGACTGGAACGGTTCCAGTCTC (forward) and GAGACTGGAACCGTTCCAGTCCTGCTGTGTCCCAT (reverse); T22N mCherry-rab7, TTCTGGAGTCGGGAAGAATTCACTCATGAACCAGT (forward) and ACTGGTTCATGAGTGAATTCTTCCCGACTCCAGAA (reverse). Cloning primers (restriction sites underlined) are as follows: EGFP-syb2 and mCherry-syb2, ATAGATCTATGTCGGCTACCGCTGCCACCG (forward) and GGGATCCTTAAGTGCTGAAGTAAACGATGATG (reverse); VAMP4 L25A–FT, TCAGAGATCTATGCCTCCCAAGTTTAAGCG (forward) and TCAACCGGTTTGGATCCAGTACGGTATTTCA (reverse); syb2-FT, ATTGTCTCGAGATGTCGGCTACCGCTGCCACCGTCC (forward) and CGTGTTGGATCCCGAGTGCTGAAGTAAACGATGATGATG (reverse); synaptophysin-FT, GCGCCTCGAGATGGACGTGGTGAATCAGCTGGTGG (forward) and GCGCCCGCGGCATCTGATTGGAGAAGGAGGTGGGC (reverse); mCherry VAMP4, GCGGCCGCGACTCTAGAT (forward) and TTATAAGGATCCAGTACGGTATTTC (reverse). Genotyping primers for VAMP4 KO animals are as follows: rev_common, GCGTAAGTCATCATCCAAACAA; Vamp4_kofor, CAAACTAGTGTTCAGATGAAG; Vamp4_wtrfor, CTAGTGTTCAGAATCAGGTGGA.

### DNA constructs

The following expression vectors were used in this study and were obtained from the indicated sources: sypHy, L. Lagnado (University of Sussex); syp-mOr2, S. Takamori (Doshisha University, Kyoto, Japan); syb2-pHluorin, G. Miesenbock (Oxford University); VAMP4 L25A–pHluorin was previously described (*5*, *14*); YFP-syntaxin1a, R. Duncan (Heriot-Watt University, Edinburgh); red fluorescent protein (RFP)–Bassoon, C. Garner (German Centre for Neurodegenerative Diseases, Berlin); iGluSnFR, K. Torok (St. George’s, University of London); psPAX2 and p-CMV-VSV-G, C. Montenegro-Venegas (Leibniz Institute for Neurobiology, Magdeburg, Germany); FUp95GW (B7) was a gift from R. Malenka, O. Schluter, and W. Xu (Addgene plasmid #74016; http://n2t.net/addgene:74016; RRID:Addgene_74016) (*61*). AP1, CHC, and scrambled shRNAs [AP1, CAAACGCATTGGCTATTTA (*39*); CHC, TCCAATTCGAAGACCAAT (*62*); scrambled, GATATGATACGATAATAAGCA (*5*)] were cloned in pSuper.neo + mCherry, which was based on pSuper.neo + GFP (oligoengine.com), modified by exchanging GFP with mCherry using Bsh TI and Bsp 1407I restriction sites. Rab7 constructs (original rab7a sequence was from S. Ferguson, University of Ottawa, Canada) were cloned into an mCherry C1 vector, which is a derivative of EGFP C1 plasmid (Clontech, Mountain View, USA), modified as described for pSuper.neo + GFP. Syb2-EGFP and syb2-mCherry were generated by amplifying the sequence of syb2 from the syb2-pHluorin plasmid and cloning it into EGFP-C1 or mCherry-C1 plasmids, respectively, using Bgl II and Bam HI restriction sites. Site-directed mutagenesis was used to generate mCherry-Q67L rab7 and T22N rab7 mutants. α-Synuclein A53T was generated via site-directed mutagenesis from wild-type α-synuclein in a pcDNA3 vector (from P. Dickson, University of Newcastle, Australia). VAMP4 L25A–FT was constructed by amplification of the VAMP4 sequence from the VAMP4-pHluorin plasmid and cloning it into pSlow-FT-N1 (plasmid 31912, Addgene) using Bgl II and Bsh TI restriction sites. syb2-FT was constructed by amplification of the syb2 sequence from the syb2-mOrange2 plasmid and cloning it into pSlow-FT-N1 using Xho I and Bam HI restriction sites. Synaptophysin-FT was constructed by amplification of the synaptophysin sequence from a mCer-synaptophysin plasmid and cloning it into pSlow-FT-N1 using Xho I and Sac II restriction sites. VAMP4-EGFP was constructed by exchanging FT from VAMP4-FT with EGFP using Bsh TI and Bsp 1407I restriction sites. A two-step process was used for the cloning of mCer VAMP4. First, the FT sequence was removed from the VAMP4-FT plasmid by a polymerase chain reaction–mediated deletion, and mCherry from mCherry N1 plasmid was inserted upstream of VAMP4 using Nhe I and Bgl II restriction sites, creating mCherry VAMP4 plasmid. Then, the VAMP4 sequence from mCherry VAMP4 plasmid was cloned into a mCer C1 plasmid using Bsp 1407I and Bam HI restriction sites. mCer syb2 was constructed by exchanging mCherry from mCherry syb2 with mCer using Bsh TI and Bsp 1407I restriction sites.

### Antibodies

The following primary antibodies were used in this study. Mouse antibodies used were against the following: actin [Western blotting (WB), 1:20,000; Sigma-Aldrich, A3854, RRID:AB_262011]; AP1γ (ICC, 1:3000; WB, 1:500; BD Biosciences, 610385, RRID:AB_397768); PSD95 (ICC, 1:500; WB, 1:500; BioLegend, 810401, AB_2564750); synaptosomal associated protein 25 (WB, 1:2000; Synaptic Systems, 111 011, RID:AB_2617076); synaptotagmin 1 (ICC, 1:2000; WB, 1:500; Abcam, ab13259, RRID:AB_299799); syntaxin1 (WB, 1:2000; Synaptic Systems, 110 011, RRID:AB_887844); synapsin 1 (ICC, 1:1000; Synaptic Systems, 106 011C2, RRID:AB_10805139); vesicle transport through interaction with t-SNAREs homolog 1A (WB, 1:10,000; BD Biosciences, 611220, RRID:AB_398752). Rabbit antibodies used were against the following: synaptotagmin 1 lumenal domain (ICC, 1:200; Synaptic Systems, 105103C5, RRID:AB_2619766); SV2A (ICC, 1:1000; Abcam, ab32942, RRID:AB_778192); synaptophysin 1 (WB, 1:1000; Synaptic Systems, 101 002, RRID:AB_1961591); syntaxin6 (WB, 1:1000; Synaptic Systems, 110 062, RRID:AB_887854); syb2 (WB, 1:1000; Abcam, ab3347, RRID:AB_2212462); VAMP4 (WB, 1:1000; Abcam, ab108051, RRID:AB_10862443); VAMP4 (ICC/IHC, 1:1000/1:500; Synaptic Systems, 136002, RRID:AB_887829); CHC (ICC, 1:50; Cell Signaling Technology, 4796, RRID:AB_10828486). The following secondary antibodies were used in this study: donkey anti-mouse Alexa Fluor 647 (ICC, 1:1000; Thermo Fisher Scientific, A31571, RRID:AB_162542); donkey anti-rabbit Alexa Fluor 568 (ICC, 1:1000; Thermo Fisher Scientific, A10042, RRID:AB_2534017); goat anti-mouse Alexa Fluor 488 (ICC, 1:1000; Thermo Fisher Scientific, A11001, RRID:AB_2534069); goat anti-rabbit Alexa Fluor 647 (ICC, 1:1000; Thermo Fisher Scientific, A21244, RRID:AB_2535812); donkey anti-rabbit immunoglobulin G (IgG) IRDye 800 CW (WB, 1:10,000; LI-COR, 92532213, RRID:AB_2715510); goat anti-mouse IgG IRDye 800 CW (WB, 1:10,000; LI-COR, 92632210, RRID:AB_621842).

### Tissue culture and transfection

Primary dissociated hippocampal cultures were prepared from either wild-type C56BL/6J E17.5 (embryonic day 17.5) embryos or *Vamp4* KO E17.5 embryos (with wild-type littermate controls) as described (*5*). Briefly, isolated hippocampi were digested in papain (10 U/ml) in Dulbecco’s phosphate-buffered saline (PBS), washed in minimal essential medium supplemented with 10% fetal bovine serum (FBS), and triturated to single-cell suspension. Cells were plated, at a desired density, on poly-d-lysine– and laminin-coated 25-mm-diameter coverslips. Neuronal cultures were maintained in Neurobasal medium supplemented with B-27, 0.5 mM l-glutamine, and penicillin and streptomycin (1%, v/v). At 3 DIV, cultures were supplemented with 1 mM cytosine β-d-arabinofuranoside to inhibit proliferation of glial cells.

Rat hippocampal cultures were prepared from E18.5 embryos. Hippocampal neurons were dissociated by trypsin-mediated digestion and mechanical trituration. Neurons were plated in medium containing 10% FBS (HyClone), 0.45% dextrose, sodium pyruvate (0.11 mg/ml), 2 mM glutamine, penicillin (100 U/ml), and streptomycin (100 mg/ml) in modified Eagle’s medium. Typically, 200,000 neurons were plated on 18-mm coverglasses coated overnight with poly-d-lysine (0.06 mg/ml) and laminin (0.0025 mg/ml) in borate buffer. After 4 hours, neurons were switched to maintenance media containing 1× B27, penicillin (100 U/ml), streptomycin (100 mg/ml), 2 mM glutamine, and 12.5 μM glutamate in Neurobasal media. Half of the medium was replaced with new medium every 4 days. Hippocampal neurons were transfected or cotransfected with two different constructs at DIV 7 and 8 using Lipofectamine 2000 (Thermo Fisher Scientific, 11668-019) following the instructions from the manufacturer, and neurons were imaged at DIV 13 to 16 as described in detail below.

### Lentiviral particle production and transduction

Lentiviral particles were produced as described (*63*). Human embryonic kidney 293T cells, grown in media supplemented with 10% FBS, were transfected with three plasmids FUp95GW, psPAX2, and p-CMV-VSV-G (molar ratio, 2:1:1) using Lipofectamine 2000. Cells were incubated with the DNA:Lipofectamine 2000 mixture for 8 hours at 37°C before the FBS medium was replaced with Neurobasal supplemented with B27, penicillin and streptomycin, and l-glutamine. The medium containing viral particles was collected at day 3, filtered using a 0.45-μm filter, and used directly for transducing neuronal cultures on DIV 10. Cultures were used at DIV 14 and 15.

### Acute slice preparation

Horizontal hippocampal slices (350 μm) were prepared from wild-type and VAMP4 KO mice (25-32 days of age of either sex). Excised brains were rapidly transferred to chilled (2° to 5°C) carbogenated sucrose-modified artificial cerebrospinal fluid (saCSF; 86 mM NaCl, 1.2 mM sodium phosphate buffer, 2.5 mM KCl, 25 mM NaHCO_3_, 25 mM glucose, 50 mM sucrose, 0.5 mM CaCl_2_, and 7 mM MgCl_2_) for 2 min and subsequently sliced in the same solution using a vibrating microtome (Leica, VT1200S). Slices were allowed to recover for 1 hour at 33°C in carbongenated standard aCSF, which contained 126 mM NaCl, 3 mM KCl, 1.2 mM sodium phosphate buffer, 25 mM NaHCO_3_, 15 mM glucose, 2 mM CaCl_2_, and 2 mM MgCl_2_.

### Electrophysiology

For recording, slices were transferred to an immersion chamber continuously perfused with standard aCSF (1 mM MgCl_2_) maintained at 32°C using an in-line Peltier heater (Scientifica, Uckfield, UK). A cut was made between CA2 and CA1 (identified as the medial termination of the stratum lucidum) to ablate recurrent activity. Whole-cell patch-clamp recordings were made from visually identified pyramidal neurons in the CA1 region using pulled borosilciate electrodes (4 to 7 megohms). The intracellular solution for evoked and intrinsic properties experiments consisted of 142 mM K-gluconate, 4 mM KCl, 0.5 mM EGTA, 10 mM Hepes, 2 mM MgCl_2_, 2 mM Na_2_ATP, 0.3 mM Na_2_GTP, and 10 mM Na_2_-creatine. For mEPSC recordings, a cesium-based intracellular solution was used consisting of 140 mM Cs-gluconate, 3 mM CsCl, 0.2 mM EGTA, 10 mM Hepes, 5 mM QX-314 chloride, 2 mM MgATP, 2 mM NaATP, 0.3 mM Na_2_GTP, and 10 mM phosphocreatine. Excitatory currents were recorded in the presence of picrotoxin (50 μM) with cells voltage-clamped at −70 mV, with the further addition of tetrodotoxin (300 nM) for mEPSC recording.

In recording protocols, intrinsic properties were recorded in current-clamp mode. All other recordings were made under voltage-clamp. Currents were low pass–filtered at 3 to 10 kHz and sampled at 10 to 20 kHz, using Clampex 10 software (pClamp 10, Molecular Devices, San Jose, USA). For evoked recordings, Schaffer collaterals were stimulated with a patch electrode (~1 to 2 megohms) filled with aCSF and positioned in the stratum radiatum, connected to an isolated constant current stimulator (Digitimer, Welwyn Garden City, UK). In all cases, the stimulus intensity was set to evoke a current of ~200 pA following a 50-μs pulse. Stimulus was delivered at either paired pulses (interval of 10 to 500 ms, pairs 30 s apart), short bursts (10 pulses at 20 Hz, 16 repeats 5 s apart), or long trains (40 Hz for 15 s, 4 repeats delivered 4 min apart). Data were analyzed offline using either the open-source Stimfit software package (intrinsic properties) or Clampfit from the pClamp 10 software suite (all EPSCs). Cells were excluded from analysis if series resistance varied by more than 20% during recording.

RRP size and its replenishment were determined using approaches described in (*64*). Briefly, the RRP was calculated by plotting the cumulative EPSC amplitude from 40-Hz 15-s trains and performing a linear regression on the last 1 s of that plot. The *y* intercept of this regression line denotes RRP size (fig. S6D). Replenishment rate is represented by the slope of the regression line. Pr was calculated as the amplitude of the first evoked EPSC divided by the effective RRP size (*45*).

### ICC, perfusion fixation, and IHC

For ICC, neurons were fixed in 4% PFA in PBS for 4 to 5 min at room temperature. Samples were blocked and permeabilized using 10% FBS, 0.1% glycine, and 0.3% Triton X-100 in PBS for 20 min and incubated with primary antibodies overnight at 4°C. Secondary antibodies were applied for 1 hour at room temperature. Coverslips were mounted on microscope slides with FluorSave reagent (Merck Millipore). Immunofluorescence intensity was quantified in circular regions of interest (ROIs), with a diameter of 1.8 μm, automatically placed over nerve terminals using the same intensity threshold in ImageJ.

Live staining with synaptotagmin 1 antibody (Syt1 Ab uptake) was performed by incubating neurons with fluorescently labeled Syt1 Ab (1:200) dissolved in a buffer containing 136 mM NaCl, 2.5 mM KCl, 2 mM CaCl_2_, 1.3 mM MgCl_2_, 10 mM glucose, and 10 mM Hepes (pH 7.4) (imaging buffer) for 30 min at 37°C. For quantitative assessments, the same antibody solutions were applied to all coverslips in the corresponding experiments.

Immunofluorescence intensity of VAMP4-EGFP and VAMP4-pHluorin (Figs. 4 and 5) was quantified in circular ROIs, with a diameter of 1.8 μm, placed manually over the nerve terminals using Time Series Analyzer in ImageJ. The nerve terminals were identified on the basis of the morphology of the axonal arbor.

The percentage of syp-mOr2, VAMP4-pHluorin, and syb2-pHluorin puncta colocalizing with PSD95-EGFP or endogenous PSD95 was determined as follows: (i) Circular ROIs with a diameter of 1.8 μm were placed manually over the nerve terminals showing accumulation of syp-mOr2, VAMP4-pHluorin, and syb2-pHluorin; (ii) the number of PSD95 puncta colocalizing with the presynaptic markers was calculated by applying an identical intensity threshold for PSD95 between the different conditions across all experiments (*63*).

For IHC, 3-month-old wild-type and VAMP4 KO mice of either sex were administered a lethal dose of sodium pentobarbital and transcardially perfused with cold PB buffer, followed by cold PFA (4% in 0.1 M PB). Brains were extracted and fixed for 24 hours in PFA at 4°C, washed with PBS, and transferred to a 30% sucrose/PBS solution for 48 hours at 4°C. Brains were embedded in tissue-freezing compound, and 50-μm coronal sections were generated using a freezing microtome. Free-floating thin sections were permeabilized for 4 to 5 hours in block solution (PBS, 10% horse serum, 0.5% bovine serum albumin, 0.5% Triton X-100, and 0.2 M glycine) and then incubated with VAMP4 primary antibody (previously precleared against VAMP4 KO tissue) diluted in block solution (1:500) overnight at 4°C. Slices were washed four to five times in PBS for 2 hours and then incubated for 3 to 4 hours with secondary antibodies (1:1000; anti-rabbit Alexa Fluor 568) and NeuroTrace green fluorescent Nissl stain (1:2000; Thermo Fisher Scientific) at room temperature. Slices were then washed four to five times in PBS for 2 hours and mounted onto glass slides using ProLong Gold antifade mountant (Thermo Fisher Scientific). Sections were imaged on a Zeiss LSM800 upright confocal microscope. The tile function within the Zeiss ZEN software was used to acquire overlapping images over the whole section, followed by the image processing function to stitch the tiles together.

### TMR-dextran uptake

TMR-dextran uptake (40 kDa) was performed at 37°C as described (*39*). Neurons, labeled with Syt1 Ab, were incubated with TMR-dextran (50 μM), dissolved in imaging buffer supplemented with 10 μM CNQX and 50 μM AP-5, and stimulated with 400 APs at 40 Hz. To remove unspecific labeling, cells were washed extensively immediately after stimulation. All live imaging of neurons was performed on an inverted Zeiss Axio Observer Z1 microscope using EC Plan-Neofluar 40× oil immersion objective (numerical aperture, 1.3) and Colibri 7 light-emitting diode light source (Zeiss). The microscope was equipped with a Zeiss AxioCam 506 camera and controlled by ZEISS ZEN 2 software. Images of neurons colabeled with Syt1 Ab and TMR-dextran were acquired using DsRed (exciter, 538 to 562 nm; beam splitter, 570 nm; emitter, 570 to 640 nm) and Cy5 (exciter, 625 to 655 nm; beam splitter, 660 nm; emitter, 665 to 715 nm) filter sets. For analysis, four to five fields of view per coverslip were acquired. The number of TMR-dextran puncta per field of view was determined using self-written macro in ImageJ. The macro uses the Maximum Entropy and Analyze Particles algorithms to threshold the image and count puncta between 0.3 and 2.5 μm. To account for differences in synapse density, the number of TMR-dextran puncta was normalized to the number of synapses labeled with Syt1 Ab (active synapses) in the same fields of view. For quantification, the number of TMR-dextran puncta was averaged per coverslip.

### Live imaging of pHluorin and mOrange2 reporters

Imaging of pHluorin and syp-mOr2 reporters was performed as described (*5*), at 37°C. Coverslips were mounted in an imaging chamber (Warner Instruments, USA, RC-21BRFS), supplied with embedded parallel platinum wires, 0.6 cm apart, for electrical stimulation. Cultures were stimulated with either 400 APs (100 mA, 1-ms pulse width) at 40 Hz or 300 APs at 10 Hz. Transfected neurons were imaged using both GFP (exciter, 450 to 490 nm; beam splitter, 495 nm; emitter, 500 to 550 nm) and DsRed filter sets, with images captured at 2-s intervals throughout. The total pHluorin-expressing pool was visualized after perfusion of alkaline imaging buffer (containing 50 mM NH_4_Cl instead of 50 mM NaCl) 180 s after termination of stimulation. This value was used for quantification of synaptic pHluorin levels, with only puncta expressing both pHluorin and syp-mOr2 selected for analysis. SVs and endolysosomes were distinguished on the basis of their axonal mobility. SVs are stable organelles, enriched in nerve terminals that may or may not participate in recycling in response to electrical stimulation, whereas endosomes are membrane organelles undergoing anterograde and retrograde long-distance trafficking in axons, which typically do not undergo fusion upon electrical stimulation. Imaging of VAMP4-EGFP (Figs. 4 and 5) was performed in living neurons in imaging buffer, with nerve terminals identified on the basis of the morphology of the axonal arbor.

For quantification of SV pools and amount of SV fusion, imaging and stimulation were performed in the presence of 1 μM bafilomycin A1 (Stratech). Treatment with 30 μM SMIFH2 (Abcam) was performed for 2 hours before and during image acquisition. Fusion of RRP SVs was triggered by stimulation with 40 APs at 20 Hz. The TRP was released by delivering 900 APs at 20 Hz. The total pHluorin-expressing SV pool was visualized after perfusion of alkaline imaging buffer as before. Fluorescent images were acquired at 0.5 Hz.

Quantification of the time-lapse series was performed using the Time Series Analyzer plugin for ImageJ. For the dual-color time-lapse series of syp-mOr2 and VAMP4/syb2 pHluorin, ROIs were placed over responding syp-mOr2 synaptic terminals and the average change in fluorescence intensity monitored in both the green and red channels. For the analysis of the extent of SV fusion and kinetics of SV endocytosis monitored by sypHy, only synapses that responded to stimulation were selected for analysis. The criteria for responding terminals in all experiments were defined by the average increase in fluorescence intensity in response to AP stimulation being ≥2% of the baseline. If required, traces were corrected for bleaching using a bleaching factor calculated from the unresponsive terminals on the same coverslip. Statistical analyses were performed in Microsoft Excel and GraphPad Prism 8.

### Imaging of FTs

Live imaging of neurons transfected with VAMP4-FT, syb2-FT, and synaptophysin-FT was performed at DIV 14 at 37°C, 7 days after transfection. Neurons were imaged using DsRed and Timer filter sets (excitation wavelength, 370 to 400 nm; beam splitter, 405 nm; emitter, 425 to 525 nm). Images were acquired first in the red channel and then in the blue channel as recommended in (*27*). The blue and red immunofluorescence intensities were measured in ROIs placed over the synaptic terminals and their ratio calculated after subtraction of background fluorescence.

### Imaging of iGluSnFR

Imaging of iGluSnFR was performed using the GFP (exciter, 450 to 490 nm; beam splitter, 495 nm; emitter, 500 to 550 nm) filter set at 37°C. Cultures were stimulated every 5 s with five consecutive trains of 10 APs (at 20 Hz). Fluorescent images were acquired every 0.3 s. Quantification of the time-lapse series was performed using the Time Series Analyzer plugin for ImageJ as described for the pHluorin imaging.

### FLIM imaging

Real-time FLIM imaging of neurons cotransfected with either mCer-VAMP4 or mCer-syb2 and YFP-syntaxin1a (mCer-syb2 alone as a negative control) was performed in imaging buffer supplemented with 10 μM CNQX and 50 μM AP-5. FLIM images were acquired on a Leica SP8 FALCON, an integrated FLIM confocal platform controlled by LAS X v. 3.5.2.18963 software equipped with a 440-nm pulsed laser (PicoQuant PDL 800-D pulsed diode laser) and a HyD single molecule detection detector. The same intensity of the pulsed laser and detector settings were used for all experiments. The images were acquired using a HC PL APO CS2 63×/1.40 oil objective. The confocal settings used for image acquisition were as follows: 512 by 512 pixels of display resolution, 8-bit dynamic range, optical zoom of 3.0, resulting in a final pixel size of 0.12 μm. Four-time line averaging was applied on frames, and scan speed was 400 Hz. The 440-nm pulsed laser was used for excitation of mCer, and the fluorescence signal was detected in the range of 451 to 520 nm. An area of interest containing a dozen of synaptic terminals was selected, and a single FLIM image was acquired at rest. A train of 800 APs at 40 Hz was then triggered offline. A second FLIM image of the same area of interest was taken 10 s after the onset of stimulation, and neurons were continuously stimulated during image acquisition. The instrument response function for the data analysis was obtained by FLIM imaging of a gold nanorod sample at the excitation wavelength. All FLIM images were exported in a PTU format using the LAS X software and analyzed offline with FLIMfit 5.1.1 (freely available at https://downloads.openmicroscopy.org/flimfit/4.11.0/).

For the analysis, synaptic ROIs were created using the segmentation tool in FLIMfit. The same ROIs were applied to the FLIM images acquired at rest (control) and during field stimulation. To compute the fluorescence lifetimes of the donor (decay time mCer τ), we applied the pixel-wise global fitting method available in FLIMfit and fitted the donor fluorescence intensity decays to a double-exponential decay model [equations 1, 3, and 5 in (*65*)]. The quality criteria for the fit were defined by the χ^2^ value being close to 1. The difference in the FRET efficiency between control and stimulated samples was calculated as: ΔFRET = τ1 mCer_control − τ1 mCer_stimulated. The pseudo-color lifetime images and matching histograms (Fig. 2, A to H) were generated using FLIMfit and were displayed over 3.0 to 4.1 ns as indicated in the figures.

The images in fig. S2G were acquired on an inverted Zeiss Axio Observer A1 microscope using a Zeiss Plan-Apochomat 40× oil objective. YFP-syntaxin1a was visualized using an excitation of 475 to 525 nm, whereas mCer-syb2 and mCer-VAMP4 were visualized using 416- to 456-nm excitation. Both were visualized using a 515-nm beamsplitter and 505- to 560-nm emission.

### Time-lapse imaging of axonal trafficking and kymograph analysis

Time-lapse imaging of neurons (DIV 13 to 15) transfected with either VAMP4-EGFP or syb2-EGFP with or without mCherry-rab7 was performed at 37°C in imaging buffer. A series of frames (120 total; frame interval, 1.4 s; total duration of time-lapse series, 2.8 min) were acquired to visualize axonal trafficking under basal conditions. Following this, neurons were stimulated with 400 APs at 40 Hz, and the motility of axonal carriers was monitored continuously during and after stimulation for an additional 2.8 min.

Kymographs were generated using the KymographClear macro toolset for ImageJ (*66*). A single distal axonal segment of 120 to 140 μm was traced per coverslip. Trajectories of mobile particles were constructed manually using the segmented line tool in KymographClear. The number of stationary carriers per axon was automatically calculated using an identical intensity threshold in KymographDirect (*66*). The number of mobile carriers, moving in anterograde and retrograde directions, was quantified as a percentage of the total number of carriers (stationary + mobile) in the corresponding axonal segments. The run length of the mobile carriers was extracted from the kymographs using KymographDirect. The percentage of VAMP4-EGFP and syb2-EGFP carriers cotrafficking with mCherry-rab7 was manually calculated from kymographs.

### Labeling endocytic pathways by HRP and electron microscopy

Hippocampal cultures were washed once with imaging buffer then stimulated with a train of 400 APs over 10 s in the presence of HRP (10 mg/ml in imaging buffer). After HRP washout, cultures were immediately fixed in 2% glutaraldehyde and 2% paraformaldehyde in 0.1 M PB for 30 min at 37°C. Unstimulated and unlabeled cultures were immediately fixed following the initial imaging media wash for baseline SV pool size measurements. After washing with 0.1 M PB, cultures were exposed to 0.1% diaminobenzidine and 0.2% H_2_O_2_ in 0.1 M PB until color developed. Cultures were washed with 0.1 M PB and stained with 1% osmium tetroxide for 30 min at room temperature. After washing in water, cultures were dehydrated using ethanol series and two final polypropylene oxide steps before being embedded in Durcupan resin. Samples were sectioned, mounted on formvar grids, and poststained with uranyl acetate and lead citrate. Sections were viewed using a JEOL TEM-1400 Plus transmission electron microscope. Nerve terminals were imaged and analyzed providing they were membrane bound and contained a pool of SVs, regardless of whether they contained HRP. Intracellular structures that were <100 nm in diameter were arbitrarily designated to be SVs, whereas larger structures were considered endosomes. HRP-labeled SVs and endosomes within each nerve terminal were manually counted using ImageJ.

### Protein biochemistry

Crude synaptosomes (P2) were prepared from the brains of age-matched wild-type and VAMP4 KO mice. Brain homogenates were prepared in tris-buffered sucrose [0.32 M sucrose, 1 mM EDTA, and 5 mM tris (pH 7.4)] and centrifuged at 1075*g* for 10 min to pellet the nuclear fraction (P1). Next, the supernatant was centrifuged at 20,200*g* for 15 min to pellet the crude synaptosomes. The P2 pellet (from individual mouse brains) was resuspended in 20 ml of Hepes-buffered Krebs (118.5 mM NaCl, 4.7 mM KCl, 1.18 mM MgSO_4_, 10 mM glucose, 1 mM sodium phosphate buffer, and 20 mM Hepes) and recentrifuged at 20,200*g* for 15 min. The supernatant was aspirated, and the pellets were frozen for subsequent use.

For quantification of protein expression using WB, synaptosomes were lysed in a buffer containing 25 mM tris-HCl (pH 7.4), 150 mM NaCl, 1 mM EGTA, 1 mM EDTA, 1% Triton X-100, and 1× protease inhibitor cocktail. SDS sample buffer [67 mM tris (pH 7.4), 2 mM EGTA, 9.3% glycerol, 12% β-mercaptoethanol, bromophenol blue, and 67 mM SDS] was added to the lysates, and samples were boiled for 10 min before separation using SDS–polyacrylamide gel electrophoresis (PAGE).

Brain lysates were prepared by homogenization in a buffer containing 25 mM tris-HCl (pH 7.4), 150 mM NaCl, 1% NP-40, 0.1% SDS, 0.5% sodium deoxycholic acid, 1 mM EDTA, 1 mM EGTA, and 1× protease inhibitor cocktail. Samples were vortexed six times while being incubated on ice for 30 min. Cell debris were removed by centrifugation (10,000*g* for 10 min), and the supernatant (whole-brain lysate) was diluted with 2× SDS sample buffer and boiled for 10 min before loading on SDS-PAGE.

Lysate pellet 2 (LP2) fractions were prepared by resuspending and homogenizing P2 synaptosomes in tris-buffered sucrose. After this, 1 M Hepes/NaOH solution (pH 7.4) was added to the suspension at 4°C. The suspension was centrifuged at 25,000*g* at 4°C for 20 min, giving LP1 and lysate supernatant (LS1). LS1 was centrifuged at 165,000*g*, which gives LP2 and LS2 fractions. LP2 was resuspended in 0.4 M sucrose.

Protein samples were resolved on SDS-PAGE and blotted onto nitrocellulose membranes. Membranes were incubated with primary antibodies overnight at 4°C and with secondary antibodies for 1 hour at room temperature. Membranes were imaged on an Odyssey 9120 Infrared Imaging System (LI-COR Biosciences) using LI-COR Image Studio Lite software (version 5.2) and analyzed using ImageJ. The integrated density of signals was measured in rectangular ROIs of an identical size set around the protein expression bands.

### Proteomic sample preparation

Synaptosome samples were subjected to acetone precipitation for 6 hours at −20°C, and the pellet was washed with chilled acetone. The pellet was air-dried and dissolved in urea lysis buffer (8 M urea in 50 mM tris-Cl and 1% sodium deoxycholate). Protein concentration for all samples was determined using the bicinchoninic acid assay (Thermo Fisher Scientific). Twenty micrograms of total protein was used for proteomic sample preparation by suspension trapping (S-Trap) (*67*), as recommended by the supplier (ProtiFi, Huntington, NY, USA). Samples were reduced with 5 mM tris (2-carboxyethyl)phosphine (Pierce) for 30 min at 37°C and subsequently alkylated with 5 mM iodoacetamide for 30 min at 37°C in the dark. Samples were acidified with 2.5 μl of 12% phosphoric acid after reduction and alkylation and prepared for proteolysis by the addition of 165 μl of S-Trap binding buffer [90% methanol in 100 mM triethylammonium bicarbonate buffer (pH 7.1)]. Acidified samples were added to S-trap microspin columns and centrifuged at 4000*g* for 1 min. Each S-trap microspin column was washed with 150 μl of S-trap binding buffer and centrifuged at 4000*g* for 1 min. This process was repeated for five washes. Twenty-five microliters of 50 mM TEAB (pH 8.0) containing sequencing-grade trypsin (1:10 ratio of trypsin:protein) was added to each sample, followed by proteolysis for 2 hours at 47°C using a thermomixer (Eppendorf). Peptides were eluted with 50 mM TEAB (pH 8.0) and centrifuged at 1000*g* for 1 min. Elution steps were repeated using 0.2% formic acid (FA) and 0.2% FA in 50% acetonitrile, respectively. The combined eluates from each sample were dried using a SpeedVac before storage at −80°C.

### Liquid chromatography–tandem mass spectrometry analysis

The peptides were reconstituted in 20 μl of 0.1% FA, vortexed, and sonicated, and 1.5 μl of each sample was injected on the mass spectrometer. Peptides were analyzed by nanoflow LC-MS/MS (liquid chromatography–tandem mass spectrometry) using an Orbitrap Fusion Lumos Tribrid mass spectrometer (Thermo Fisher Scientific) coupled to a Dionex Ultimate RSLC 3000 system. Samples were injected on a 100-μm inside diameter × 5-mm trap (Thermo Trap Cartridge, 5 mm) and separated on a 75 μm–by–50 cm nano-LC column (EASY-Spray LC columns, #ES803). All solvents used were high-performance liquid chromatography or LC-MS grade (Millipore). Peptides were loaded for 5 min at 10 μl/min using 0.1% FA and 2% acetonitrile in water. The column was conditioned using 100% buffer A [0.1% FA and 3% dimethyl sulfoxide (DMSO) in water], and the separation was performed on a linear gradient from 0 to 35% buffer B (0.1% FA, 3% DMSO, 16.9% water, and 80% acetonitrile) over 120 min at 300 nl/min. The column was then washed with 90% buffer B for 5 min and equilibrated 10 min with 100% buffer A in preparation for the next analysis. Full MS scans were acquired from mass/charge ratio (*m/z*) of 400 to 1600 at resolution 120,000 at *m/z* 200, with a target automatic gain control of 4 × 10^5^ and a maximum injection time of 50 ms. MS/MS scans were acquired in IonTrap mode using a fixed high-energy collision dissociation energy of 30% and resolution 15,000 using a Top 20 method, with a target AGC of 2 × 10^5^ and a maximum injection time of 50 ms. The MS/MS triggering threshold was set at 5 × 10^3^, and the dynamic exclusion of previously acquired precursor was enabled for 45 s.

### Proteomic data analysis

Peptide identification was performed by submitting the raw data to MaxQuant software (version 1.6.6.0). MS/MS spectra were searched against the Uniprot SwissProt database including isoforms (updated January 2019) using a false discovery rate of 1%. The maximum of missed cleavage was set to 2 using trypsin/P enzyme. Carbamidomethylation (C) was set as fixed modification, and acetylation (Protein N term), oxidation (M), deamination (NQ) were set as variable modifications. Label-free quantification was performed where match between runs was used with match window time (0.7) and alignment time window (20 min). Group comparisons were performed using Student’s *t* test using a *P* value (<0.05) for truncation using Perseus software (version 1.6.6.0). The *P* value–significant proteins were filtered for differentially expressed proteins by applying a fold change of 1.5. The differentially expressed proteins were subjected to bioinformatic analysis using Enrichr (*68*) software for enrichment of GO terms. Raw proteomic data were deposited on PRIDE (www.ebi.ac.uk/pride/; PXD021437—Control of synaptic vesicle PR via VAMP4 targeting to endolysosomes).

### Statistical analysis

Statistical analysis was performed using GraphPad Prism 8.4.3. No statistical methods were used to predetermine sample sizes, and no randomization procedures were applied. Statistical tests were applied on the basis of the distribution of the datasets measured using D’Agostino-Pearson normality test. Normally distributed data are presented as means or means ± SEM, whereas non-Gaussian datasets are shown as median ± interquartile range. Significance was set as nonsignificant *P* > 0.05, *P < 0.05, **P < 0.01, ***P < 0.001, and ****P < 0.0001. Mann-Whitney (two-tailed), Wilcoxon matched-pairs signed-rank (two-tailed), and Kruskal-Wallis with Dunn’s post hoc tests were used to compare non-Gaussian datasets. Student’s *t* test (two-tailed) and analyses of variance (ANOVAs) followed by Tukey’s post hoc test were used to compare normally distributed datasets. Two-way ANOVA was used to determine genotype effects and interaction for multigroup comparisons where appropriate. Information about sample sizes, statistical tests used to calculate *P* values, and the numeric values of the results are specified in the figure legends and table S2.

## SUPPLEMENTARY MATERIALS

Supplementary material for this article is available at http://advances.sciencemag.org/cgi/content/full/7/18/eabf3873/DC1

## Appendix

View/request a protocol for this paper from *Bio-protocol*.
